# CellGO: a novel deep learning-based framework and webserver for cell-type-specific gene function interpretation

**DOI:** 10.1093/bib/bbad417

**Published:** 2023-11-22

**Authors:** Peilong Li, Junfeng Wei, Ying Zhu

**Affiliations:** State Key Laboratory of Medical Neurobiology, MOE Frontiers Center for Brain Science, Institutes of Brain Science and Department of Neurosurgery, Huashan Hospital, Fudan University, Shanghai 200032, China; State Key Laboratory of Medical Neurobiology, MOE Frontiers Center for Brain Science, Institutes of Brain Science and Department of Neurosurgery, Huashan Hospital, Fudan University, Shanghai 200032, China; State Key Laboratory of Medical Neurobiology, MOE Frontiers Center for Brain Science, Institutes of Brain Science and Department of Neurosurgery, Huashan Hospital, Fudan University, Shanghai 200032, China

**Keywords:** cell-type-specific pathway analysis, gene functional analysis, artificial neural network, disease risk genes, Gene Ontology

## Abstract

Interpreting the function of genes and gene sets identified from omics experiments remains a challenge, as current pathway analysis tools often fail to consider the critical biological context, such as tissue or cell-type specificity. To address this limitation, we introduced CellGO. CellGO tackles this challenge by leveraging the visible neural network (VNN) and single-cell gene expressions to mimic cell-type-specific signaling propagation along the Gene Ontology tree within a cell. This design enables a novel scoring system to calculate the cell-type-specific gene-pathway paired active scores, based on which, CellGO is able to identify cell-type-specific active pathways associated with single genes. In addition, by aggregating the activities of single genes, CellGO extends its capability to identify cell-type-specific active pathways for a given gene set. To enhance biological interpretation, CellGO offers additional features, including the identification of significantly active cell types and driver genes and community analysis of pathways. To validate its performance, CellGO was assessed using a gene set comprising mixed cell-type markers, confirming its ability to discern active pathways across distinct cell types. Subsequent benchmarking analyses demonstrated CellGO’s superiority in effectively identifying cell types and their corresponding cell-type-specific pathways affected by gene knockouts, using either single genes or sets of genes differentially expressed between knockout and control samples. Moreover, CellGO demonstrated its ability to infer cell-type-specific pathogenesis for disease risk genes. Accessible as a Python package, CellGO also provides a user-friendly web interface, making it a versatile and accessible tool for researchers in the field.

## INTRODUCTION

The prevalence of high-throughput technologies has greatly accelerated the discovery of genes with specific functions and their associations with diseases [1–5]. A genome-wide association study (GWAS) can reveal hundreds of thousands of disease risk genes [6–13], and a single RNA-seq experiment may detect from one to thousands of differentially expressed genes (DEG) [14–20]. However, the challenge remains in mechanistic interpretation of these discoveries.

The common strategy to gain mechanistic insights from genes is pathway analysis [21, 22]. In spite of various forms, the underlying concept is to find a statistic to measure the pathway ‘activities’ given a gene set. The pathway ‘activity’ can be assessed simply by quantifying the number of genes shared between the test gene set and the genes within the pathway. Subsequently, the pathways deemed ‘active’ are chosen if the number of overlapped genes is significantly greater than the background [23]. This approach is called over-representation analysis (ORA) and is adopted by many popular tools, such as PANTHER [24], gProfileR [25–27], DAVID [28] and clusterProfiler [29]. However, ORA relies on the assumption that all genes make independent and equal contributions to pathways, which may not always hold true in biological systems. To overcome this limitation, functional class scoring (FCS) has been proposed, for example, gene set enrichment analysis (GSEA) [30] and gene set variation analysis (GSVA) [31]. FCS computes pathway activities by aggregating gene-level scores [32, 33], such as expression fold changes (FC), which reflect the contributions of genes to each pathway. However, FCS requires scores for all genes, making it challenging when only a gene set is available. Moreover, neither ORA nor FCS takes into account the interrelationship between genes. Pathway topology-based methods, such as NetGSA [34], TopoGSA [35], CTpathway [36], LPIA [37] and PathNet [38], have been developed to address this limitation by integrating the interconnections between genes within pathways, potentially improving performance over previous approaches. Nevertheless, few of the three categories of methods consider cross talk between pathways.

Moreover, the biological context in which pathways is active (e.g. specific tissues or cell types) can provide critical information for mechanistic explanations [22]. Unfortunately, the aforementioned methods often fail to account for this vital information. Recently, several approaches have been developed to address it. For instance, scMappR [39] combines bulk RNA-seq deconvolution with ranked ORA to obtain cell-type-specific pathways enriched in DEG. Vision [40] and PAGODA [41] aggregate gene expression in single cells to calculate gene-set activities, and identify cell-type-specific active pathways by ranking these activities across cell types. However, these tools are tailored for analyzing single-cell RNA-seq (scRNA-seq) data and rely on such data as input. There is a pressing need for a tool capable of convenient cell-type-specific gene functional analysis based on user’s selected genes of interest.

The behavior of a cell is determined by the complex interplay of its constituent pathways. Through inter-pathway cross talk, the gene activity can propagate from one part of the network to others, and the coordination of the entire network determines the development and maintenance of cellular phenotypes. To understand collaboration of genes and pathways in cells, it is necessary to simulate the propagation of signals throughout the entire network, capturing both intra-pathway gene interactions and inter-pathway cross talk. Existing databases, such as Gene Ontology [42, 43] (GO), provide pathway details and their connections, with each of the three biological domains (Biological Process (BP); Molecular Function (MF); Cellular Component (CC)) organizing the directed acyclic graph that resembles the hierarchical organization within a cell. Recent advances in deep learning algorithms have led to the development of novel methods for modeling the hierarchical structure of subsystems [44]. In particular, VNNs have shown promise in learning the signaling propagation within a cell, enabling the dissection of underlying pathways related to specific cellular phenotypes because of its ‘visible’ nature [45, 46].

We developed CellGO for cell-type-specific pathway analysis. CellGO integrates the single-cell RNA expression data and the VNN model that emulates the hierarchy of GO terms to capture cell-type-specific signatures, intra-pathway gene connections and inter-pathway cross talk. CellGO proposes a novel framework that identifies cell-type-specific active pathways given a single gene or a gene set. Its gene-set mode additionally outputs significantly activated cell types, and for a certain cell type, the prioritized pathway communities and most active genes (MAGs) within active pathways. We tested the performance of CellGO against existing tools in identifying cell-type-specific active pathways across multiple experimental data sets [14–18] and extended its applicability to a vast collection of 71 publicly available single-cell data sets of human and mouse brains [47–75]. CellGO is accessible as a python package cellgopy (https://github.com/FduZhuLab/cellgopy) and through a user-friendly web interface (http://www.cellgo.world and https://zhuy-lab.fudan.edu.cn/CellGO#).

## METHODS

### Design of CellGO

**Figure 1:**
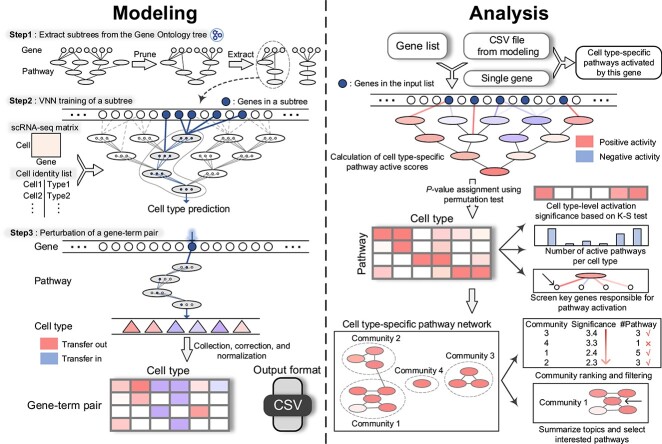
CellGO workflow. CellGO predicts cell-type-specific active pathways of a single gene or a gene set through a modeling phase (left) and an analysis phase (right). In the modeling phase, CellGO first prunes the GO tree and extracts subtrees starting from each leaf term (step 1). Next, CellGO utilizes the single-cell gene expression matrix to learn cell-type labels by training VNNs based on subtrees (step 2). It then scores the cell-type-specific activity of each gene-term pair as GTSs by quantifying prediction changes in cell-type labels when simulating gene perturbation. Cell-type-specific GTSs are reported as a CSV file for subsequent analysis. In the analysis phase, CellGO employs a novel pathway activation measuring system to infer cell-type-specific active pathways in the single-gene mode, and a four-level hierarchy of biological interpretation in the gene-set mode.

CellGO is designed to identify cell-type-specific active pathways given a single gene or a gene set, operating in two phases: a modeling phase and an analysis phase (Figure 1).

#### The modeling phase

During the modeling phase, the cell-type-specific signaling transduction process within a cell is simulated using single-cell gene expression data to predict cell-type labels through the training of a VNN with a structure that mirrors the hierarchy of GO terms. The VNN used in CellGO is a non-fully connected feedforward neural network, where neurons of a GO term receive inputs from neurons of its direct child terms and the genes present in this term but not in its child terms. To capture the multifunction of each term, CellGO represents each term with multiple neurons ranging from 20 to 50. CellGO constructs a global VNN using all GO terms. Additionally, CellGO constructs a subtree for each leaf term (a term without any child terms), consisting of the leaf term and all its parental terms that are directly or indirectly connected to it, and learns cell-type labels using this subtree. The gene-term score (GTS), which represents the paired active score between a gene and the term it directly connects to, is determined by simulating the gene expression to 0 and measuring the resulting alteration of cell identities. The modeling phase outputs a comma-separated values (CSV) file containing cell-type-specific GTSs of all gene-term pairs.

#### The analysis phase

The analysis phase takes the CSV file from the modeling phase, along with either a single gene or a gene set as input. CellGO can predict the cell types and cell-type-specific pathways significantly activated by a single gene by calculating empirical *P*-values for GTSs. For a gene set, gene-pathway paired active scores (GPPASs) are calculated based on GTSs. GTSs are assigned as GPPASs for directly connected gene-pathway pairs. When a gene and a pathway are indirectly connected, the GPPAS is computed by averaging the GTSs of the pathway’s all direct and indirect children that are directly connected to the gene. The cell-type-specific pathway active score (ctPAS) is obtained by summing the GPPASs of all genes in both the pathway and the set. To identify cell-type-specific active pathways, CellGO compares ctPASs with the empirical distribution calculated by randomly sampling the same number of genes.

The analysis phase offers several modules for mechanistic interpretation of the query gene set. First, it can select cell-type-specific active pathways based on empirical *P*-values and *z*-scored ctPASs. Second, it can identify the cell type(s) most activated by the enquiry gene set by either ranking the number of active pathways or by comparing the distribution of GTSs with the background utilizing Kolmogorov–Smirnov (K–S) test across cell types. Third, CellGO can construct the network of cell-type-specific active pathways and report top communities enriched with active pathways, by incorporating the random walk with restart (RWR) algorithm [76] and the community partition algorithm. In this study, we manually summarize the topic of a community to maximally cover the semantics of pathways within the community. Lastly, CellGO allows for the selection of the MAG for each active pathway based on GPPASs.

### Workflow of CellGO

The modeling phase includes steps 1–3, and the analysis phase includes steps 4–7. Detailed descriptions of each step are in Supplementary Text S1–S2.

#### Step 1: Processing of ontologies and extraction of subtrees

We downloaded the GO ontology and annotation data from https://doi.org/10.5281/zenodo.6799722, and assigned genes to GO terms based on species-specific GO annotations. Then we pruned the species-specific annotated GO graph and extracted subtrees from the pruned GO graph. For each leaf term, we regarded it and all of its parental terms, the parent–child relations between these terms, and the annotated genes of each term, as a subtree.

#### Step 2: Training the VNN of each subtree

The VNN was inspired by Dcell [45], and the differences of the VNN between CellGO and Dcell are discussed in Supplementary Text S3. For each subtree, CellGO constructs and trains a VNN whose architecture mirrors the inter-pathway parent–child relations and intra-pathway gene annotations. We denote our training data set as $D=\left\{\left({X}_1,{y}_1\right),\left({X}_1,{y}_1\right),\dots, \left({X}_N,{y}_N\right)\right\}$, where *N* is the number of samples (or cells). For each sample *i*, ${X}_i\in{R}^G$ denotes the gene expression, represented as a vector of continuous values on *G* genes, and ${y}_i\in{R}^C$ denotes the cell identity, represented as a one-hot vector of binary values on *C* classes (or cell types). ${y}_{ic}=1$ if sample *i* is annotated to class *c*, otherwise ${y}_{ic}=0$. The multidimensional state of each term (or pathway) *p*, denoted by the output vector ${O}_i^{(p)}$, is defined by a nonlinear function of the contribution of its annotated genes ${g}^{(p)}$ (if present) and the states of all of its first-order children ${\mathrm{child}}^{(p)}$, concatenated in the input vector ${I}_i^{(p)}$:


(1) \begin{equation*} {Z}_i^{(p)}=\mathrm{Tanh}{\left(\mathrm{Linear}\left({X}_{ig}\right)\right)}_{g\in{g}^{(p)}} \end{equation*}

(2) \begin{equation*} {I}_i^{(p)}=\mathrm{Concatenate}\left({Z}_i^{(p)},{\left\{{\mathrm{O}}_i^{\left({p}^{\prime}\right)}\right\}}_{p^{\prime}\in{\mathrm{child}}^{(p)}}\right) \end{equation*}

(3) \begin{equation*} {O}_i^{(p)}=\mathrm{Batch}\ \mathrm{Norm}\left(\mathrm{Tanh}\left(\mathrm{Linear}\left({I}_i^{(p)}\right)\right)\right) \end{equation*}

Let ${L}_O^{(p)}$denote the length of ${O}_i^{(p)}$ determined by


(4) \begin{align*} {L}_O^{(p)}=&\min \left(50,\max \left(20,\left[0.3\ast \mathrm{number}\ \mathrm{of}\ \mathrm{genes}\ \mathrm{annotated}\ \mathrm{in}\ p \right.\right.\right. \nonumber \\ &\left.\left.\left. \mathrm{and}\ \mathrm{all}\ \mathrm{of}\ \mathrm{its}\ \mathrm{children}\right]\right)\right) \end{align*}

We performed the training process by minimizing the objective function:


(5) \begin{align*} & \frac{1}{N}{\sum}_{i=1}^N\Big(\mathrm{Loss}\left(\mathrm{Softmax}\left(\mathrm{Tanh}\left(\mathrm{Linear}\left({O}_i^{(r)}\right)\right)\right),{y}_i\right)\nonumber \\ & +\alpha{\sum}_{p\ne r}\left(\mathrm{Loss}\left(\mathrm{Softmax}\left(\mathrm{Tanh}\left(\mathrm{Linear}\left({O}_i^{(p)}\right)\right)\right),{y}_i\right)\right)+\lambda{\left\Vert W\right\Vert}_2 \end{align*}

Here, Loss is the cross-entropy loss function. *r* is the top-level term of the subtree. The parameter $\alpha$ (=0.3) balances these two contributions. $\lambda$ (=0.001) is an *l*_2_ norm regularization factor.

#### Step 3: Scoring cell-type-specific activities of gene-term pairs

Let $gp\in{GP}^{(v)}$ denote all gene-term paired annotation relations within subtree *v*, the raw score of a gene-term pair $gp$ in class *c* is determined by


(6) \begin{equation*} {S}_{gp,c}^{(v)}=-\left(\begin{array}{@{}c@{}}\mathrm{c}\mathrm{hange}\ \mathrm{of}\ \mathrm{sample}\ \mathrm{number}\ \mathrm{predicted}\ \mathrm{as}\ \mathrm{c}\ \mathrm{by}\ \mathrm{r}\ \\{}\mathrm{when}\ \mathrm{setting}\ \mathrm{the}\ \mathrm{expression}\ \mathrm{of}\ \mathrm{g}\ \mathrm{in}\ \mathrm{p}\ \mathrm{to}\ 0\end{array}\right) \end{equation*}

We next eliminated the class-specific background bias by


(7) \begin{equation*} {S}_{gp,c}^{(v)}\leftarrow{S}_{gp,c}^{(v)}-\mathrm{median}\left({\left\{{S}_{g^{\prime}{p}^{\prime},c}^{(v)}\right\}}_{g^{\prime}{p}^{\prime}\in{GP}^{(v)}}\right) \end{equation*}

We next corrected these scores according to the classification accuracy of *r*:


(8) \begin{equation*} {\mathrm{accuracy}}_{\mathrm{random}}=\frac{\max \left(\mathrm{number}\ \mathrm{of}\ \mathrm{samples}\ \mathrm{per}\ \mathrm{class}\ c\right)}{\mathrm{number}\ \mathrm{of}\ \mathrm{all}\ \mathrm{samples}} \end{equation*}

(9) \begin{equation*} {\mathrm{size}\ \mathrm{factor}}^{(v)}=\max \left(0,\frac{{\mathrm{accuracy}}_r^{(v)}-{\mathrm{accuracy}}_{\mathrm{random}}}{1-{\mathrm{accuracy}}_{\mathrm{random}}}\right) \end{equation*}

(10) \begin{equation*} {S}_{gp,c}^{(v)}\leftarrow{S}_{gp,c}^{(v)}\ast{\mathrm{size}\ \mathrm{factor}}^{(v)} \end{equation*}

Here, ${\mathrm{size}\ \mathrm{factor}}^{(v)}=0$ if the accuracy of *r* is less than the accuracy of classifying all samples to the class with the maximum number. Let *V* denote the collection of every subtree; we computed GTSs without consideration of *v*:


(11) \begin{equation*} {S}_{gp,c}=\mathrm{mean}\left({\left\{{S}_{gp,c}^{(v)}\right\}}_{v\in V}\right) \end{equation*}

Let *GP* denote the collection of every gene-term pair. We balanced the power of positive and negative scores:


(12) \begin{equation*} {S}_{gp,c}\leftarrow{S}_{gp,c}\ast \frac{\sum_{g^{\prime}{p}^{\prime}\in GP}\left(-{S}_{g^{\prime}{p}^{\prime},c}\right)\ast \mathbb{I}\left({S}_{g^{\prime}{p}^{\prime},c}\le 0\right)}{\sum_{g^{\prime}{p}^{\prime}\in GP}\left({S}_{g^{\prime}{p}^{\prime},c}\right)\ast \mathbb{I}\left({S}_{g^{\prime}{p}^{\prime},c}>0\right)},\forall{S}_{gp,c}>0 \end{equation*}

Lastly, we normalized these scores to the interval $\left[-1,1\right]$:


(13) \begin{equation*} {S}_{gp,c}\leftarrow \frac{S_{gp,c}}{\max \left(\mathrm{abs}\left({\left\{{S}_{gp,c}\right\}}_{gp\in GP}\right)\right)} \end{equation*}

We defined the *P*-value of a cell-type-specific GTS by


(14) \begin{equation*} P-{\mathrm{value}}_{gp,c}=\frac{\sum_{g^{\prime}{p}^{\prime}\in GP}\ \mathbb{I}\left({S}_{gp,c}<{S}_{g^{\prime}{p}^{\prime},c}\right)}{\mathrm{number}\ \mathrm{of}\ \mathrm{all}\ \mathrm{gene}-\mathrm{term}\ \mathrm{pairs}} \end{equation*}

#### Step 4: Assigning cell-type-specific P-values to pathways given a gene list

Given a set of genes denoted by $g\in{G}^{(u)}$. Let ${GP}^{(p)}$denote the collection of all gene-term pairs which involve term *p* or any one of its child terms, and let ${G}^{(p)}$ denote all annotated genes of term *p* and all of its child terms. CellGO next computed ctPASs through summing GPPASs:


(15) \begin{equation*} {A}_{gp,c}=\mathrm{mean}\left({\left\{{S}_{g{p}^{\prime},c}\right\}}_{g{p}^{\prime}\in{GP}^{(p)}}\right),\forall g\in{G}^{(p)} \end{equation*}

(16) \begin{equation*} {A}_{p,c}^{(u)}={\sum}_{g\in \left({G}^{(u)}\bigcap{G}^{(p)}\right)}{A}_{gp,c} \end{equation*}

Here, ${A}_{gp,c}$ denotes the cell-type-specific GPPAS of gene *g* in pathway *p*, if *p* or its children contain *g*. ${A}_{p,c}^{(u)}$ denotes the ctPAS of *p* by summarizing GPPASs of genes in both ${G}^{(u)}$ and ${G}^{(p)}$. We further assigned the $P-{\mathrm{value}}_{p,c}^{(u)}$ to ${A}_{p,c}^{(u)}$ based on permutation test.

#### Step 5: Analysis of cell-type level activation

We used the python package SciPy [77] to perform K–S test to evaluate the cell-type-level significance on a given gene list:


(17) \begin{align*} P-{\mathrm{value}}_c^{(u)}=&\mathrm{K}\hbox{--} \mathrm{S}\ \mathrm{test}\left({\left\{{S}_{gp,c}\right\}}_{g\in{G}^{(u)}},{\left\{{S}_{gp,c}\right\}}_{g\in \left(G-{G}^{(u)}\right)}, \right. \nonumber \\ & \left. \mathrm{alternative} =^{`}{\mathrm{less}}^{{\prime}}\right) \end{align*}

#### Step 6: Cell-type-specific pathway network analysis

We screened cell-type-specific active pathways by $P-{\mathrm{value}}_{p,c}^{(u)}\le \mathrm{Active}P$ (default 0.01) and took them as seed nodes of RWR to explore high-affinity nodes in BP. We then divided these high-affinity nodes to determine pathway communities using the python package graph-tool (DOI: 10.6084/m9.figshare.1164194.v14). Let $p\in{hP}_{\mathrm{BP},t}^{(c)}$ denote pathways in community *t* of cell type *c* in BP; the community-level significance is determined by


(18) \begin{equation*} {\mathrm{Significance}}_{\mathrm{BP},t}^{(c)}=\mathrm{mean}\left({\left\{\min \Big(4,-{\log}_{10}\left(P-{\mathrm{value}}_{p,c}^{(u)}\right)\Big)\right\}}_{p\in{hP}_{\mathrm{BP},t}^{(c)}}\right) \end{equation*}

We defined communities with significance greater than $-{\log}_{10}\left(\mathrm{Active}P\right)$ (default 2) as active (or significant) communities. We filtered out communities with a low number of pathways by the average number of pathways contained by significant communities.

#### Step 7: ORA-based pathway analysis in CellGO

We used SciPy [77] to perform Fisher’s exact test to evaluate the pathway-level significance on a given gene list:


(19) \begin{align*} & P-{\mathrm{value}}_p^{(u)}={\mathrm{Fisher}}^{\prime}\mathrm{s}\ \mathrm{exact}\ \mathrm{test} \nonumber \\& \left(\begin{array}{@{}c@{}}\left[\begin{array}{cc}\mathrm{length}\left({G}^{(u)}\bigcap{G}^{(p)}\right)& \mathrm{length}\left({G}^{(u)}-{G}^{(u)}\bigcap{G}^{(p)}\right)\\{}\mathrm{length}\left({G}^{(p)}\right)& \mathrm{length}\left(G-{G}^{(p)}\right)\end{array}\right],\\{}\ \mathrm{alternative}={}^{`}{\mathrm{greater}}^{{\prime}}\end{array}\right) \end{align*}

## RESULTS

**Figure 2:**
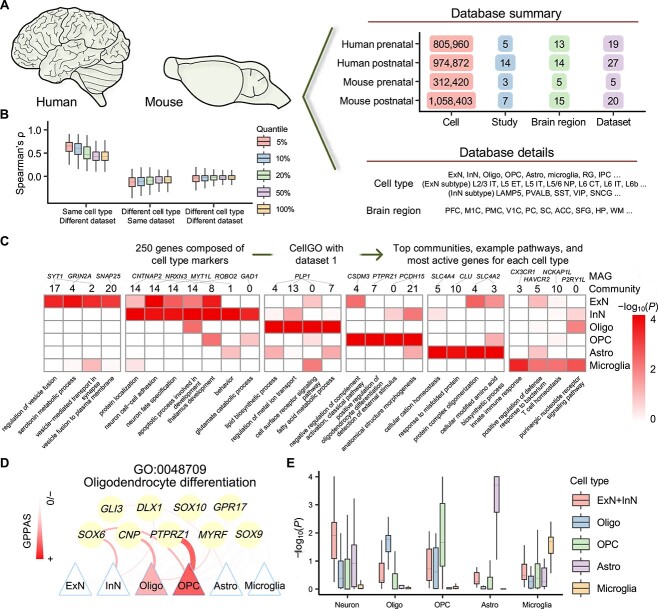
Application of CellGO to capture cell-type-specific signatures with built-in data sets. (**A**) Overview of brain cell data sets inbuilt in CellGO. The numbers of cells, studies, brain regions and data sets are shown in the upper right. Representative cell types and brain regions are listed in the lower right. The full names of cell types are listed in Supplementary Table S1. (**B**) Boxplot showing pairwise Spearman’s rank correlation coefficients across GTSs of the same cell type from different data sets (left), GTSs of different cell types from the same data set (middle) and GTSs of different cell types from different data sets (right), calculated using 27 human postnatal data sets. Spearman’s correlations calculated using different quantiles of gene-term pairs were displayed. For example, 5% indicates that the correlations were calculated on gene-term pairs with GTSs ranked in the top 5% in either group. The horizontal middle line of each boxplot denotes the median. The bounds of the box represent 0.25 quantile (*Q*_1_) and 0.75 quantile (*Q*_3_), respectively. The upper whisker is the minimum of the maximum value and *Q*_3_ + 1.5*IQR, where IQR = *Q*_3_ − *Q*_1_. The lower whisker is the maximum of the minimum value and *Q*_1_ − 1.5*IQR. (**C**) Top four communities, example pathways in these communities, and MAGs of these pathways for each cell type identified by CellGO testing 250 cell-type markers using data set 1. Heatmap colors denote the cell-type-specific log-transformed *P*-values of example pathways in the top four communities per cell type. The community index and the MAG of each pathway are shown above the heatmap. (**D**) Activation map showcasing genes in the oligodendrocyte differentiation pathway across different cell types. The thickness and colors of lines denote GPPASs (see Methods). (**E**) Box plot showing the distribution of cell-type-specific log-transformed *P*-values from CellGO for predefined cell-type-specific pathways. ‘ExN + InN’ in the legend denotes the maximum value of the minus log-transformed *P*-values of ExN and InN.

To conduct a comprehensive performance assessment and showcase the practical application of CellGO, we trained CellGO on 71 single-cell data sets separately [47–75] (Supplementary Table S1 and Supplementary Text S4), comprising over 3 million cells from 47 prenatal and postnatal brain regions and 34 cell types (Figures 2A and S1).

### CellGO’s reproducibility and robustness

We first tested the prediction accuracy of CellGO on cell-type classification based on data set 1 (Supplementary Figure S2). The global VNN, which matched the entire GO graph, achieved a cell-type classification accuracy of 99.6% according to the results repeating 5-fold cross-validation for five times. Additionally, we trained models based on 4168 VNNs matched with subtrees that included a median of 10 pathways and 99 genes. These models reached a median accuracy of 78.7% in cell-type classification.

As a stochastic learning algorithm, a feedforward neural network produces different optimized parameters each time it is run. To assess the reproducibility of CellGO’s neural networks, we performed the modeling phase four times on data set 1. We found high correlations of GTSs between replicates of the same cell type (Supplementary Figure S3). Specifically, considering gene-term pairs ranking in the top 5% in either of the two replicates, the median Spearman’s *ρ* achieved 0.904, and 0.785 for all gene-term pairs. Conversely, we observed very low correlations of GTSs between different cell types within (median Spearman’s *ρ* = −0.0537) and between (median Spearman’s *ρ* = −0.0391) replicates, indicating distinct gene-term activation patterns among cell types. Similar patterns were observed in human postnatal data sets (Figure 2B), albeit with lower correlations for both top 5% gene-term pairs (median Spearman’s *ρ* = 0.665) and all gene-term pairs (median Spearman’s *ρ* = 0.433) between different data sets of the same cell type. These lower correlations could be partially attributed to differences in cell types across brain regions and batch effects between data sets. Overall, these results indicated that CellGO generates replicable and robust GTSs for downstream analysis.

### Accurate deconvolution of cell-type-specific signatures from mixed cell-type markers by CellGO

To assess the ability of CellGO to accurately identify cell-type-specific pathways, we assembled a query gene set consisting of the top 50 marker genes for each cell type [78] (Supplementary Table S2). The significant pathways identified by Fisher’s exact test, using each cell type’s marker genes independently, were considered as ground truth of predefined cell-type-specific pathways (Supplementary Table S3 and Supplementary Text S5). Our analysis using the assembled set identified top communities enriched with active pathways with distinct semantics in different cell types (Figure 2C and Supplementary Table S4). In addition, CellGO assigned more significant *P*-values for predefined cell-type-specific pathways in their corresponding cell types (Figure 2E). These results were corroborated by another data set (Supplementary Figure S4 and Supplementary Table S5).

Moreover, CellGO can identify MAGs in cell-type-specific active pathways. In excitatory neurons (ExN), the gene *SYT1* that encodes a membrane protein was identified as the MAG in pathways, such as regulation of vesicle fusion and vesicle-mediated transport in synapse, belonging to top communities (Figure 2C). In oligodendrocytes, we indicated *PLP1*, a gene encoding a transmembrane proteolipid protein, as the MAG in lipid biosynthetic process and regulation of metal ion transport. Notably, CellGO determines MAGs of the same pathway in a cell-type-specific manner. For instance, CellGO identified *PTPRZ1* as the MAG of oligodendrocyte differentiation in oligodendrocyte precursor cells (OPC) (Figure 2D), consistent with its role in maintaining OPC in an undifferentiated state [79]. In contrast, CNP, a marker of late-phase OPC, was inferred as the MAG of the same pathway in oligodendrocytes, aligning with its function in the retention of the myelin sheath [80].

Together, we demonstrated CellGO’s capability to identify distinct enriched pathways in different cell types, along with their corresponding driver genes, from a gene list.

### Functional inference of gene knockouts and comparative experiments

**Figure 3:**
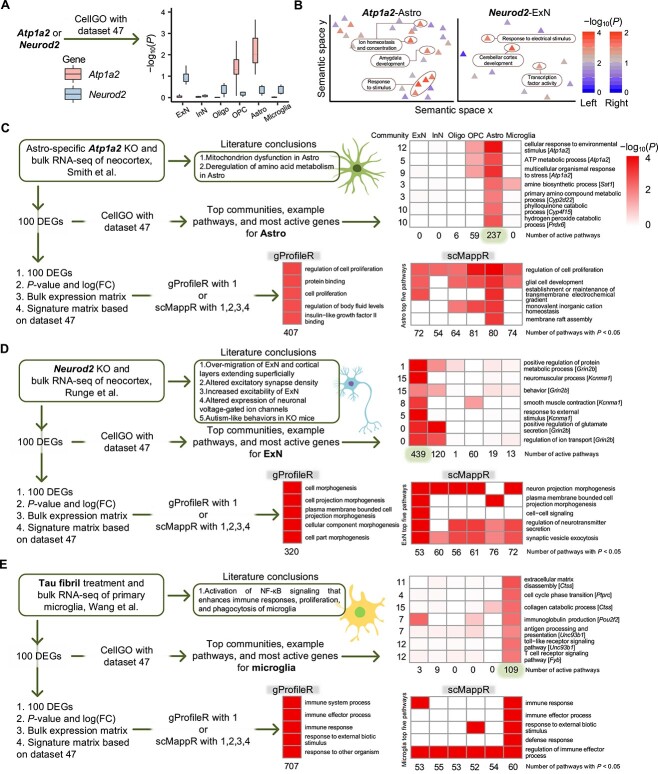
Benchmarking CellGO’s performance on experimental data sets. (**A**) Boxplot illustrating the distribution of cell-type-specific log-transformed *P*-values, mimicking *Atp1a2* and *Neurod2* KO, using single-gene mode based on data set 47 (see Methods). The boxplot displays the median as the horizontal middle line, the 0.25 quantile (*Q*_1_) and 0.75 quantile (*Q*_3_) as the bounds of the box, and the upper and lower whiskers based on the minimum of the maximum value and *Q*_3_ + 1.5*IQR, and the maximum of the minimum value and *Q*_1_ − 1.5*IQR, respectively. (**B**) Semantic map of pathways predicted to be perturbed in *Atp1a2* (left) and *Neurod2* (right) KOs, respectively, using single-gene mode. The colors of triangles denote the log-transformed *P*-values of pathways directly connected to *Atp1a2* in astrocytes (left) or log-transformed *P*-values of pathways directly connected to *Neurod2* in ExN (right). The most significant pathways are elliptically labeled and annotated with their names. Embedding of pathways into the semantic space is described in Supplementary Text S11. (**C**–**E**) Comparison of results obtained from CellGO, gProfileR and scMappR when analyzing DEG lists from (C) Smith *et al*., (D) Runge *et al*. and (E) Wang *et al*. The main conclusions reported in the original studies are listed as ground truth. Heatmap colors represent log-transformed *P*-values for CellGO and FDR-adjusted *P*-values for gProfileR and scMappR. In CellGO heatmaps, example pathways in the top five communities of the most active cell type are plotted. The number of active pathways (*P*-value < 0.01) per cell type is displayed underneath the heatmap, and the MAG of each pathway is provided next to the pathway name. The community index of each pathway is shown on the left side of the heatmap. The gProfileR heatmaps depict the top five significant pathways, with the number of significant pathways (FDR-adjusted *P* < 0.05) shown underneath the heatmap. The scMappR heatmaps present the top five significant pathways in the same cell types as the corresponding CellGO heatmaps, with the number of significant pathways (FDR-adjusted *P* < 0.05) of each cell type labeled underneath the heatmap.

The performance of CellGO on cell-type-specific pathway analysis was assessed using gene knockout (KO) data sets [14, 15, 17, 18] (Supplementary Table S6 and Supplementary Text S6). First, we applied CellGO to examine *Atp1a2* using single-gene analysis based on the model trained on a mouse primary visual cortex data set (data set 47). Our analysis revealed astrocytes as the most affected cell type by *Atp1a2* perturbation, displaying the most significant *P*-values of GTSs compared with other cell types (Figure 3A). Pathways with the highest GTSs in astrocytes were related to ion homeostasis and stimulus responses (Figure 3B and Supplementary Table S7), matching experimental studies associating *Atp1a2* with astrocyte-mediated K and Ca ion transport and LPS-induced inflammation [81–83]. In contrast, pathways with the lowest GTSs in astrocytes were related to neuron-dependent processes, including learning, behaviors and neurotransmitter uptake. As analysis of a single gene depends on its annotation in the GO database, we extended our analysis to the top 100 DEG between astrocyte-specific *Atp1a2* KO and WT mice to gain detailed insights [14]. CellGO revealed the largest number of active pathways in astrocytes. Noteworthily, three out of the top five communities of astrocytes represented topics related to metabolic processes (Supplementary Table S8). More specifically, ATP metabolic process, amine biosynthetic process and primary amino compound metabolic process were discovered in these communities (Figure 3C), with *Atp1a2*, *Sat1*, a gene encoding an enzyme involved in the polyamine metabolism, and *Cyp2d22*, a gene encoding an enzyme that participates in wide metabolic and biosynthetic processes, as the MAGs in these pathways, respectively. These findings align with a study [14] indicating *Atp1a2* deletion in astrocytes leads to alterations of transcript levels of metabolic enzymes and metabolite levels of serine and glycine, as well as impairments in mitochondrial functions.

For comparison, we analyzed the top 100 DEG using the widely used pathway analysis tool, gProfileR [25–27], and the cell-type-specific pathway enrichment tool, scMappR [39] (Supplementary Text S7). The top enriched pathways identified by gProfileR included regulation of cell proliferation, protein binding and regulation of body fluid levels (Figure 3C and Supplementary Table S8). Notably, gProfileR discovered 407 enriched pathways in BP and MF domains, a larger number than astrocyte-active pathways in CellGO. Pathways revealed by gProfileR included some with semantics that align with the conclusions of experimental studies [14], such as cellular modified amino acid catabolic process and potassium-transporting ATPase activity, although these pathways are ranked below the top 20 in gProfileR results. scMappR identified the largest number of significant pathways in OPC, and displayed a lower level of cell-type specificity in enriched pathways compared with CellGO. Specifically, 84% (357 out of 425) of the pathways enriched in a certain cell type were also enriched in other cell types in scMappR, whereas only 8% (24 out of 302) of active pathways were found to be shared across multiple cell types in CellGO. Moreover, few experimentally verified pathways were reported as significant in astrocytes by scMappR (Supplementary Figure S5A and Supplementary Text S8).

Next, we utilized CellGO to predict the effect of *Neurod2* KO on cell-type-specific pathways. Single-gene analysis revealed *Neurod2* primarily affected ExN (Figure 3A), with most influenced pathways related to electrical stimulus responses, cerebellar cortex development and transcription factor activity (Figure 3B). Expanding the analysis to the top 100 DEG between *Neurod2* KO and WT mice [15], CellGO identified ExN as the cell type with the highest activation (Figure 3D). The top communities in ExN represented topics related to nervous and muscle system processes, secretion, behaviors, stimulus responses and transport (Supplementary Table S8), and specific pathways within them are shown in Figure 3D, with *Grin2b* predicted as the MAG of multiple pathways. Other communities significantly enriched with ExN-active pathways possessed various topics associated with cell death, cell differentiation, anatomical structure morphogenesis, ion homeostasis and membrane potential (Supplementary Figure S6A). These findings are consistent with reported functions of *Neurod2* on ExN differentiation and maturation in the neocortex [15, 84]. KO of *Neurod2* leads to excessive migration of ExN, resulting in alterations of cortical layer sizes and positionings [15]. Additionally, *Neurod2* depletion induces dysregulated expression of ion channels, elevation of ExN action potentials in response to depolarizing current injections as well as the manifestation of autism-like behaviors [15].

In contrast to CellGO, gProfileR highlighted top enriched pathways related to cell morphogenesis (Figure 3D and Supplementary Table S8). scMappR, on the other hand, identified astrocytes as the cell type with the largest number of significant pathways, and only one out of the top five enriched pathways in ExN showed cell-type specificity. Furthermore, CellGO reported a significantly higher number of pathways associated with experimentally validated functions than scMappR in ExN, providing further evidence of the accuracy of CellGO in uncovering cellular and molecular mechanisms (Supplementary Figure S5B).

The consistency between CellGO’s predictions and experimental validations was substantiated by analyzing *Adnp* KO [17] and *Wwox* knockdown [18] data sets (Supplementary Figure S6B–C and Supplementary Text S9). Furthermore, we demonstrated CellGO’s implementation in analyzing DEG from comparative experiments between chemical treatment and normal control. Our analysis of primary microglia treated with tau fibrils [16] revealed microglia exhibited the highest activation (Figure 3E), with the top communities of microglia representing topics related to immune and metabolic processes, extracellular structure organization and cell cycle (Supplementary Table S8). These communities included pathways such as extracellular matrix disassembly, cell cycle phase transition, immunoglobulin production and toll-like receptor signaling pathway (Figure 3E), with *Ctss*, *Ptprc*, *Pou2f2* and *Unc93b1* identified as the MAGs in these pathways, respectively. These findings align with experimental studies [16] revealing tau activates the NF-κB signaling that enhances microglial immune responses, proliferation and phagocytosis. Both gProfileR and scMappR analyses also identified top enriched pathways related to immune responses (Figure 3E and Supplementary Table S8). While microglia possessed the largest number of significant pathways in scMappR, other cell types also enriched a set number of experimentally validated pathways (Supplementary Figure S5C).

Overall, these results demonstrated that CellGO outperformed existing tools in identifying functionally relevant pathways with higher cell-type specificity.

### Cell-type-specific pathway analysis of disease risk genes

Large-scale GWASs are pivotal in identifying risk genes for various diseases [6–13]. However, understanding the pathogenic mechanisms remains challenging, despite efforts to infer disease-associated cell types and biological processes [85–87]. We applied CellGO to investigate the cell-type-level pathogenesis of Alzheimer’s disease (AD), autism spectrum disorder (ASD) and Parkinson’s disease (PD), by analyzing risk genes using data sets from the primarily affected brain regions of these disorders.

**Figure 4:**
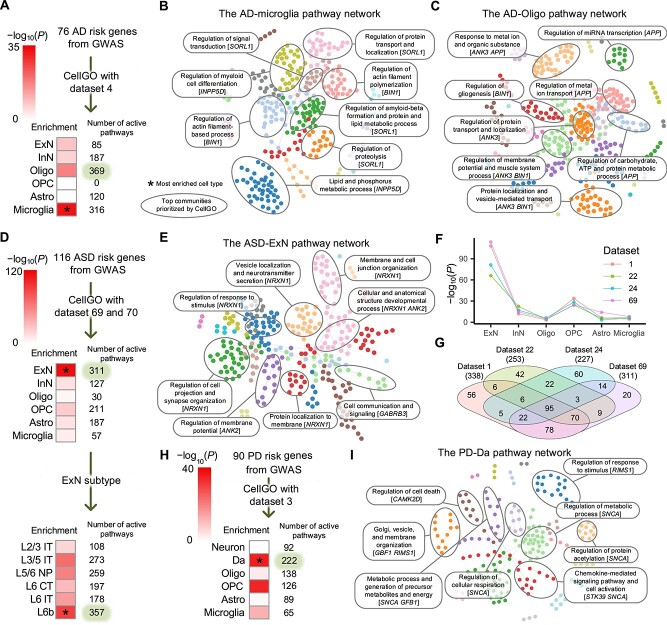
Application of CellGO to disease risk genes. (**A**–**C**) Results of CellGO analysis performed on 76 AD risk genes using data set 4. (A) The cell-type enrichment of AD-active pathways. Heatmap colors denote the log-transformed *P*-values of cell types based on K–S test of GTSs (see Methods). The asterisk denotes the most enriched cell type. The number of active pathways (*P* < 0.01) of each cell type is shown on the right side of the heatmap. (B, C) The network of AD-active pathways in (B) microglia and (C) oligodendrocytes. The pathways are colored by communities, with the top eight communities elliptically labeled and annotated with topics. MAGs of pathways with close semantics to the topic of communities are labeled. (**D**–**G**) Results of CellGO analysis performed on 116 ASD risk genes. (D) The cell-type and ExN subtype enrichment of ASD risk genes based on data set 69, respectively, and 70. (E) The network of ASD-active pathways in ExN. (F) Cell-type enrichment of pathways activated by ASD risk genes using four different PFC data sets. Log-transformed *P*-values of K–S test based on GTSs were plotted. (G) Venn diagram showing the numbers of shared ASD-associated ExN-active pathways between four PFC data sets. The total number of ExN-active pathways per data set is shown in parentheses. (**H**) The cell-type enrichment of pathways activated by 90 PD risk genes using data set 3. (**I**) The network of PD-associated pathways in Da.

CellGO was first used to analyze 76 risk genes of AD [6] (Supplementary Table S9) using a human entorhinal cortex (EC) data set (data set 4), as EC exhibits the earliest histological alterations in AD [88]. Our analysis identified microglia and oligodendrocytes as the most closely associated cell types [59], in terms of both the number of active pathways and cell-type-level enrichment using K–S test (Figure 4A). The top communities of microglia represented topics related to proteolysis, amyloid-beta formation, lipid and protein metabolic processes, signal transduction and myeloid cell differentiation (Figure 4B and Supplementary Table S10), consistent with reported findings in AD patients where abnormal microglial responses to amyloid-beta are associated with the accumulation of amyloid plaques [89], and altered protein and lipid metabolism in microglia with AD pathology [90–92]. In addition, we determined *SORL1* as the MAG of pathways across multiple top communities (Figure 4B), aligning with its function in amyloid-beta destruction [93]. The top communities of oligodendrocytes revealed biological processes distinct from those in microglia (Figure 4C), characterized by topics related to metal ion transport and responses, membrane potential and gliogenesis. These findings corroborate results revealed by single-cell analysis that genes related to ion transmembrane transport and membrane potential were down-regulated in oligodendrocytes of AD patients [94]. Furthermore, we discovered *APP*, a gene encoding the amyloid precursor protein, as the MAG of pathways in communities associated with metal ion transport, protein metabolic processes and organic substance responses (Figure 4C). APP is known to play an essential role in AD, where accumulation of amyloid-beta peptides from proteolytic cleavage of the amyloid precursor protein is the factor in dysregulation of the ion transport process and the development of AD [95, 96].

Next, we applied CellGO to study 116 ASD risk genes [7–10] using a prefrontal cortex (PFC) data set (data set 69). We found that ExN, particularly deep-layer subtypes, exhibited the highest level of activation [97] (Figure 4D). ExN’s top communities possessed topics associated with synapse and membrane organization, vesicle localization, membrane potential, signaling and developmental processes (Figure 4E). Similar results were found in the ExN subtype L6b (Supplementary Figure S7). These findings are in line with studies that revealed the impairment of synaptic development and functions in ASD [47, 98]. Furthermore, we uncovered *NRXN1* as the MAG of pathways in communities associated with synapse organization, stimulus responses and neurotransmitter secretion (Figure 4E). This finding aligns with its function in the formation of synaptic contacts and efficient neurotransmission [98–100]. Furthermore, the cell-type enrichment and the active pathways identified in ExN showed a high overlap across multiple PFC data sets (Figure 4F–G and Supplementary Table S11). Specifically, over 93% of active pathways identified in data set 69 were also present in the other three data sets, indicating the robustness of CellGO.

For PD [11–13], we focused on the substantia nigra, the brain region most affected by PD [101, 102]. We found dopaminergic neurons (Da) showed the highest activation by risk genes [101, 102] (Figure 4H). The top communities of Da were linked to topics related to the generation of energy, cell respiration, cell death, cell activation, chemokine-mediated signaling pathways, and vesicle and membrane organization (Figure 4I). These topics correspond to impaired mitochondrial homeostasis and defective cellular respiration reported as factors causing Da loss in PD [103, 104]. Moreover, *SNCA*, the gene encoding alpha-synuclein, was identified as the MAG of pathways in multiple communities (Figure 4I), aligning with the role of alpha-synuclein in PD, where its accumulation in Da leads to mitochondrial dysfunction and cell death [103, 105].

Further training CellGO using a peripheral blood mononuclear cell data set [106] for cell-type-specific functional analysis of risk genes associated with systemic lupus erythematosus [107] confirmed its competence for applications in organs other than the brain (Supplementary Figure S8 and Supplementary Text S10). Together, our findings collectively underscored CellGO’s capacity to unveil the cell-type-specific pathogenesis of polygenic disorders.

### Web interface of CellGO

**Figure 5:**
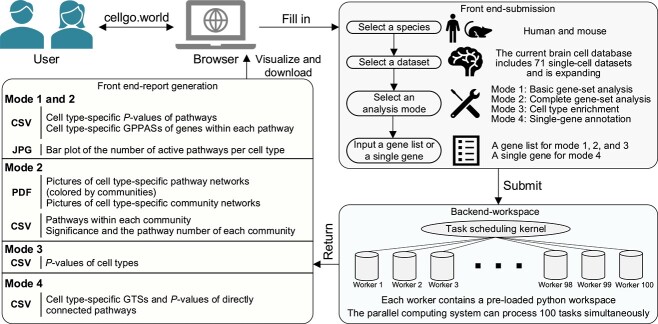
CellGO web interface. Pipeline illustration of the CellGO web interface. Users start by inputting the necessary parameters to submit an analysis request (upper right). The submission is then processed by the parallel computing system (lower right), and the results are subsequently delivered to the front end for visualization and download (left).

We built a website to provide an interactive interface of CellGO (Figure 5). This web interface provides access to pretrained models from 71 human and mouse brain single-cell data sets and offers four analysis modes. The basic gene-set analysis mode reports cell-type-specific *P*-values for pathways and cell-type-specific GPPASs for genes, with a bar plot indicating the number of active pathways per cell type. The complete gene-set analysis mode enhances the basic mode by performing pathway network analysis, providing visualizations for the network of cell-type-specific pathway communities (Supplementary Figure S9) and information on each community. The cell-type enrichment mode calculates *P*-values for cell types based on a set of gene names. The single-gene annotation mode reports cell-type-specific GTSs and *P*-values for pathways directly connected to the queried gene.

## DISCUSSION

CellGO represents a functionally novel tool for cell-type-specific pathway analysis that takes into account the biological context of pathway activation. By simulating the cell-type-specific signal propagation of genes through the hierarchical structure of the GO tree, CellGO can predict cell-type-specific pathways activated by a single gene and provide a comprehensive hierarchy of biological insights for a gene set.

The core strength of CellGO lies in its ability to consider complex interactions across genes and pathways within a cell, which is made possible through the integration of the single-cell RNA expression data and the VNN that mirrors the functional organization of a cell. This design accurately reflects the signaling propagation within a cell, spanning from genes to pathways and finally to cell identities. As a result, it enables a holistic scoring system to precisely gage the cell-type-specific activities of functional pathways given a single gene or a gene set. This new design significantly enhances CellGO’s performance in terms of cell-type-specific active pathways and its ability to identify driver genes, surpassing the capabilities of its comparators.

Furthermore, CellGO provides community-level significance that considers the cooperative contribution of topologically adjacent pathways in the GO graph to capture biological relevance by reducing the redundancy of synonymous pathways. Additionally, CellGO supports the inference of highly active genes within key pathways, providing insights into molecular origins.

It is noteworthy that CellGO may be influenced by batch effects between scRNA-seq data sets. Therefore, in our website, we provide models pretrained on each data set individually. Advancements in batch correction methods may overcome this limitation, allowing CellGO to be trained using merged data from multiple resources and expanding its applicability to cross-organ analysis. Another limitation of CellGO is its current inability to accept gene-level statistics as input, such as FC obtained from differential expression analysis. Future versions of CellGO can address this limitation by considering FC during the gene expression perturbation in the modeling phase, rather than simply setting gene expression to zero. In addition, the current VNN incorporates only GO structures, which could be expanded to include other gene–gene network information, such as protein–protein interaction networks, to better capture signaling transmission within cells.

Furthermore, we are actively working on expanding the availability of pretrained models from various organs and species on our web interface. We plan to accept user submissions and provide regular updates to make our web tool more user-friendly and accessible to a broader audience.

**Key Points**

- CellGO is a novel tool to perform cell-type-specific functional analysis for a single gene or a gene set. It integrates the single-cell gene expressions with a ‘visible’ neural network that emulates the hierarchical structure of GO terms to capture the intricate interplay between genes, gene-pathways, pathways and cell types.
- CellGO develops a holistic scoring system that identifies cell-type-specific active pathways given a single gene or a gene set.
- CellGO facilitates community-level and gene-level analysis of cell-type-specific active pathways, enabling identification of top communities enriched with active pathways and key genes driving the pathway activity.
- CellGO has been implemented on a vast collection of 71 publicly available single-cell data sets and is accessible through a python package and a multi-mode and high-concurrency web tool.

## Supplementary Material

Supplementary_Table_1_bbad417

Supplementary_Table_2_bbad417

Supplementary_Table_3_bbad417

Supplementary_Table_4_bbad417

Supplementary_Table_5_bbad417

Supplementary_Table_6_bbad417

Supplementary_Table_7_bbad417

Supplementary_Table_8_bbad417

Supplementary_Table_9_bbad417

Supplementary_Table_10_bbad417

Supplementary_Table_11_bbad417

Supplementary_Table_12_bbad417

Supplementary_Information_bbad417

## Data Availability

URLs for downloading 71 processed scRNA-seq data sets originally published by 29 studies are reported in Supplementary Table S1. Seventy-one reprocessed scRNA-seq data sets with cell labels that organize our currently proposed brain cell database are available at http://www.cellgo.world. The bulk expression matrix for the Smith *et al*. *Atp1a2* KO data set was downloaded from https://www.ncbi.nlm.nih.gov/geo/query/acc.cgi?acc=GSE145102. The bulk expression matrix for the Runge *et al*. *Neurod2* KO data set was downloaded from https://www.ncbi.nlm.nih.gov/geo/query/acc.cgi?acc=GSE110491. The bulk expression matrix for the Wang *et al*. tau fibril data set was downloaded from https://www.ncbi.nlm.nih.gov/geo/query/acc.cgi?acc=GSE198013. We release our additional data at https://doi.org/10.6084/m9.figshare.23571762.v1, including all necessary files for generating all results of the paper.

## CODE AVAILABILITY

All the functions of CellGO have been integrated into a python package cellgopy available at https://github.com/FduZhuLab/cellgopy. A Zenodo repository of the software is available here: https://doi.org/10.5281/zenodo.8078074. We also provide a tutorial for installation and operation of this python package at http://www.cellgo.world. The web interface for interactive execution of CellGO analysis is available at http://www.cellgo.world. Code for generating all results of the paper is available at https://doi.org/10.6084/m9.figshare.23571762.v1.

## Notes

**Peilong Li** is currently a PhD candidate at Institutes of Brain Science, Fudan University, China. His research interests include development of bioinformatics methods.

**Junfeng Wei** is currently a postgraduate at Institutes of Brain Science, Fudan University, China. His research interests include development of bioinformatics methods.

**Ying Zhu** received her PhD degree in neurobiology from Yale University in 2014. She is currently a full professor in Institutes of Brain Science, Fudan University, China. Her research interests include gene decoding of brain development and evolution, as well as development of bioinformatics methods.
