# Supplementary material for: CellGO: a novel deep learning-based framework and webserver for cell-type-specific gene function interpretation
Source: Brief Bioinform. 2023 Nov 22;25(1):bbad417. doi: 10.1093/bib/bbad417 (PMC10790717; doi:10.1093/bib/bbad417)
Supplement: Supplementary_Information_bbad417 [file supplementary_information_bbad417.pdf]

Supplementary Text

1 Gene Ontology

The Gene Ontology [1,2] depicts a tree-like hierarchical graph where each GO term is a node and edges represent parent-child relations between GO terms. We downloaded the ontology file 'go-basic.obo' from https://doi.org/10.5281/zenodo.6799722. This file is the basic version of the ontology, which guarantees the GO graph to be acyclic. We also downloaded the human- and mouse-specific GO annotation files ('goa_human.gaf.gz' and 'goa_human_isoform.gaf.gz' for human; 'mgi.gaf.gz' for mouse) from https://doi.org/10.5281/zenodo.6799722 to species-specifically assign gene products to GO terms. The ontology and annotation data have the release version 2022-7-1 (DOI: 10.5281/zenodo.6799722).

2 Model details of CellGO

CellGO consists of two phases: (i) The modeling phase includes steps 1-3, developed for scoring cell type-specific activities of gene-term pairs to enable cell type-specific pathway analysis for a single gene, and (ii) the analysis phase includes steps 4-7, developed for comprehensive cell type-specific pathway analysis that works for a gene set. Detailed descriptions of each step are as follows.

Step 1: Processing of ontologies and extraction of subtrees

We used the python package goatools [3] v1.0.15 to import the GO graph into the workspace to make it operable and assigned genes to GO terms based on species-specific GO annotations. Then we pruned the species-specifically annotated GO graph according to the following steps: (i) A GO term with less than six annotated genes was deleted, and all genes within it were passed to its first-order parental terms. This procedure was performed from bottom to up to ensure a term is deleted only when it has no child terms. (ii) We deleted the Cellular Component (CC) branch due to its small number of terms. Only the Biological Process (BP) and Molecular Function (MF) branches were retained. After this step, the human- and mouse-specifically annotated GO graph retained 9,870 and 9,979 terms, respectively. (iii) For each non-leaf term (a term with at least one child term), we removed annotated genes within it which simultaneously be annotated in its child terms. (iv) For each non-leaf term, we removed all genes within it if more than 100 genes were annotated. This step avoids the excessive influence of a single term on the global graph. After the above processing of ontologies, we next extracted subtrees from the pruned GO graph. Concretely, for each leaf term with no more than 100 genes, we exported it and all of its parental terms, the parent-child relations between these terms, and the annotated genes of each term as a single file with a specified format. A total of 4,168 and 4,230 subtrees were extracted from the human- and mouse-specifically pruned GO graph, respectively. This part has been coded into a function to facilitate processing other ontology files.

Step 2: Training the VNN of each subtree

The VNN was initially inspired by Dcell [4], whose neural network architecture matched the hierarchical structure of the GO graph. The differences of the VNN between CellGO and Dcell are discussed in Supplementary Text S3. Given a subtree including interrelations between terms and annotated genes within each term, CellGO constructs and trains a VNN whose architecture mirrors the inter-pathway parent-child relations and intra-pathway gene annotations. We denote our input training dataset as $D = \{(X_1, y_1), (X_1, y_1), \ldots, (X_N, y_N)\}$, where N is the number of samples (or cells). For each sample i, $X_i \in R^G$ denotes the gene expression, represented as a vector of continuous values on G genes, and $y_i \in R^C$ denotes the cell identity, represented as a one-hot vector of binary values on C classes (or cell types). $y_{ic} = 1$ if sample i is annotated to class c, otherwise $y_{ic} = 0$. If an annotated gene does not exist in the single-cell expression matrix, we pad the expression of this gene with zero. The multidimensional state of each term (or pathway) p, denoted by the output vector $O_i^{(p)}$, is defined by a nonlinear function of the contribution of its annotated genes $g^{(p)}$ (if present) and the states of all of its first-order children $child^{(p)}$, concatenated in the input vector $I_i^{(p)}$:

$$Z_i^{(p)} = Tanh(Linear(X_{ig}))_{g \in g^{(p)}} \quad (1)$$

$$I_i^{(p)} = Concatenate\left(Z_i^{(p)}, \left\{O_i^{(p')}\right\}_{p' \in child^{(p)}}\right) \quad (2)$$

$$O_i^{(p)} = Batch\ Norm(Tanh(Linear(I_i^{(p)}))) \tag{3}$$

Here, $Z_i^{(p)}$ is the contribution vector of annotated genes of p with length 10. *Concatenate* returns a single vector that is the concatenation of all input vectors. Let $L_O^{(p)}$ denote the length of $O_i^{(p)}$, representing the number of values in the state of p and determined by

$$L_O^{(p)} = \min(50, \max(20, [0.3 * \text{number of genes annotated in } p \text{ and all of its children}])) \tag{4}$$

In equation (3), the parameters of *Linear* give the ability to capture the complex biological signals of p. *Tanh* and *Batch Norm* reduce the impact of extreme values and scale differences of weights to accelerate the training speed. We performed the training process by minimizing the objective function:

$$\frac{1}{N}\sum_{i=1}^{N} (Loss(Softmax(Tanh(Linear(O_i^{(r)}))), y_i) + \alpha \sum_{p \neq r} (Loss(Softmax(Tanh(Linear(O_i^{(p)}))), y_i)) + \lambda \| W \|_2 \tag{5}$$

Here, *Loss* is the cross-entropy loss function, and r is the top-level term of the subtree. The top-level term can only be one of 'biological_process' (GO:0008150) and 'molecular_function' (GO:0003674). *Linear* in equation (5) denotes the linear function transforming the multi-dimensional vector $O_i^{(p)}$ into a vector of continuous values with length C. Every term is optimized to predict the cell identities by itself. The parameter α (=0.3) balances these two contributions. λ (=0.001) is an l_2 norm regularization factor. We optimized the objective function using ADAM, and the batch size was selected as one-third of the sample number of the class with the minimum number. We trained each subtree-matched VNN separately using 15 epochs, given its strong learning ability and fast convergence, which also reduced overfitting. We downsampled classes with over 10,000 samples and oversampled classes with less than 500 samples at each epoch (Supplementary Table S1).

Step 3: Scoring cell type-specific activities of gene-term pairs

After achieving a well-trained VNN corresponding to a subtree v, CellGO scores the cell type-specific activities of gene-term pairs within it. Let $gp \in GP^{(v)}$ denote all gene-term paired annotation relations within v, and the raw score of a gene-term pair gp in class c is determined by

$$S_{gp,c}^{(v)} = -\begin{pmatrix}\text{change of sample number predicted as } c \text{ by } r \\ \text{when setting the expression of } g \text{ in } p \text{ to } 0\end{pmatrix} \tag{6}$$

Intuitively, the top-level term r is the parent of all terms in the subtree except itself, which may better respond to the influence of the perturbation of g in p on the whole subtree. When the predicted number of c is decreased then this score is positive and vice versa. We noticed that the impact of the perturbation was transmitted upward along the network architecture to r, which simulated the transmission process of biological signals within the hierarchical functional structure of a cell. We next eliminated the class-specific background bias by

$$S_{gp,c}^{(v)} \leftarrow S_{gp,c}^{(v)} - \text{median}\left(\left\{S_{g'p',c}^{(v)}\right\}_{g'p' \in GP^{(v)}}\right) \tag{7}$$

This step allows these scores to reflect relative activity differences between gene-term pairs, and removes the internal bais of the VNN which may cause every score is extreme in a certain class. We next corrected these scores according to the classification accuracy of r:

$$accuracy_{random} = \frac{\max(\text{number of samples per class } c)}{\text{number of all samples}} \tag{8}$$

$$size\ factor^{(v)} = \max\left(0, \frac{accuracy_r^{(v)} - accuracy_{random}}{1 - accuracy_{random}}\right) \tag{9}$$

$$S_{gp,c}^{(v)} \leftarrow S_{gp,c}^{(v)} * size\,factor^{(v)} \tag{10}$$

Here, $size\,factor^{(v)}$ measures the predictive ability of r. $size\,factor^{(v)} = 1$ if the classification accuracy of r equals 1, and $size\,factor^{(v)} = 0$ if the accuracy of r is less than the accuracy of classifying all samples to the class with the maximum number. We performed this step because if genes annotated in v do not have any cell type-specific signatures, the VNN at this time is not predictable, hence these scores should be rejected. To reduce the time-consuming of the modeling phase, the classification accuracy of r is replaced by the classification accuracy for the training dataset rather than the accuracy based on five-fold cross-validation. Let V denote the collection of every subtree. Since a gp may appear in multiple subtrees, we next computed gene-term scores without consideration of v:

$$S_{gp,c} = \text{mean}\left(\left\{S_{gp,c}^{(v)}\right\}_{v \in V}\right) \tag{11}$$

Let GP denote the collection of every gene-term pair. We further balanced the power of positive and negative scores by

$$S_{gp,c} \leftarrow S_{gp,c} * \frac{\sum_{g'p' \in GP}\left(-S_{g'p',c}\right) * \mathbb{I}\left(S_{g'p',c} \leq 0\right)}{\sum_{g'p' \in GP}\left(S_{g'p',c}\right) * \mathbb{I}\left(S_{g'p',c} > 0\right)}, \forall S_{gp,c} > 0 \tag{12}$$

Lastly, we normalized these scores to the interval $[-1, 1]$ by

$$S_{gp,c} \leftarrow \frac{S_{gp,c}}{\max\left(\text{abs}\left(\left\{S_{gp,c}\right\}_{gp \in GP}\right)\right)} \tag{13}$$

So far, we have completed the estimation of cell type-specific gene-term scores. To facilitate storage and transfer, we exported them as a CSV file. We further defined the P-value of a cell type-specific gene-term score by

$$P\text{-value}_{gp,c} = \frac{\sum_{g'p' \in GP} \mathbb{I}\left(S_{gp,c} < S_{g'p',c}\right)}{\text{number of all gene-term pairs}} \tag{14}$$

We ran the above pipeline on 71 datasets (Supplementary Table S1) separately to obtain 71 CSV files for downstream analysis. Dataset 1, which includes 25,639 cells and six cell types, spent about 35 hours using one NVIDIA GeForce RTX 2080 Ti GPU.

Step 4: Assigning cell type-specific P-values to pathways given a gene list

Given a set of genes denoted by $g \in G^{(u)}$. Let $GP^{(p)}$ denote the collection of all gene-term pairs which involve term p or any one of its child terms, and let $G^{(p)}$ denote all annotated genes of term p and all of its child terms. CellGO next computed cell type-specific pathway active scores (ctPASs) through summing gene-pathway paired active scores (GPPASs):

$$A_{gp,c} = \text{mean}\left(\left\{S_{gp',c}\right\}_{gp' \in GP^{(p)}}\right), \forall g \in G^{(p)} \tag{15}$$

$$A_{p,c}^{(u)} = \sum_{g \in \left(G^{(u)} \cap G^{(p)}\right)} A_{gp,c} \tag{16}$$

Here, $A_{gp,c}$ denotes the cell type-specific GPPAS of gene g in pathway p, if p or its children contain g. $A_{p,c}^{(u)}$ denotes the ctPAS of p by summarizing GPPASs of genes in both $G^{(u)}$ and $G^{(p)}$. The most active gene in a cell type is defined as the gene with the largest cell type-specific GPPAS. We noticed that a higher GPPAS indicating a more positive and larger contribution to pathway activation. We next generated approximate null distribution of ctPASs by randomly sampling B (default 100,000) gene sets $G_K^{(b)}, \forall b \in \{1, \ldots, B\}$ with K genes

in each:

$$E_{g,c} = \text{mean}\left(\{S_{gp,c}\}_{gp\in GP}\right) \tag{17}$$

$$E_{c,K}^{(b)} = \sum_{g\in G_K^{(b)}} E_{g,c}\,,\ \forall b\in\{1,\ldots,B\} \tag{18}$$

$$Distribution_{c,K} = \left\{E_{c,K}^{(b)}\right\}_{b=1}^{B} \tag{19}$$

Here, $E_{g,c}$ denotes the cell type-specific gene active score of gene g without consideration of p, a universal approximation of $A_{gp,c}$ in equation (15). $E_{c,K}^{(b)}$ denotes the cell type-specific active scores without consideration of p. $Distribution_{c,K}$ denotes the null distribution of ctPASs with consideration of gene number K. Let $K_p^{(u)}$ denote the number of genes contained by both $G^{(u)}$ and $G^{(p)}$, we assigned the P-value to $A_{p,c}^{(u)}$ by

$$P\text{-value}_{p,c}^{(u)} = \frac{\sum_{b=1}^{B}\mathbb{I}\left(E_{c,K_p^{(u)}}^{(b)} > A_{p,c}^{(u)}\right)}{B} \tag{20}$$

In order to make $A_{p,c}^{(u)}$ comparable between different pathways and cell types, we calculated the z-scored ctPAS as the output value of the ctPAS by

$$z\text{-}score_{p,c}^{(u)} = \frac{A_{p,c}^{(u)} - \text{mean}\left(Distribution_{c,K_p^{(u)}}\right)}{\text{SD}\left(Distribution_{c,K_p^{(u)}}\right)} \tag{21}$$

Step 5: Analysis of cell-type level activation

We used the python package scipy [5] v1.5.4 to perform Kolmogorov-Smirnov (K-S) test to evaluate the cell type-level significance on a given gene list:

$$P\text{-value}_{c}^{(u)} = \text{K-S test}\left(\{S_{gp,c}\}_{g\in G^{(u)}}, \{S_{gp,c}\}_{g\in (G-G^{(u)})}, \text{alternative} = \text{'less'}\right) \tag{22}$$

Step 6: Cell type-specific pathway network analysis

We screened cell type-specific active pathways by $P\text{-value}_{p,c}^{(u)} \le \text{Active}P$ (default 0.01) and took them as seed nodes of random walk with restart (RWR) to explore high-affinity nodes in the pruned GO graph. Since BP and MF are two non-interconnected domains, we implemented RWR on the two domains separately. Let $p\in P_{\text{BP}}^{(c)}$ denote active pathways of cell type c in BP, we then went on 25 travels starting from each of them, and each travel included ten steps. The first step of each travel was at the starting node. Each of the other nine steps had a 10% probability of returning to the starting and a 90% probability of randomly entering one neighbor (one parent or one child) of the current node. We then selected high-affinity nodes according to the times each node be traveled. The number of high-affinity nodes was defined as [1.1 * number of seed nodes]. It should be noted that high-affinity nodes allow the inclusion of seed nodes.

Let $p\in hP_{\text{BP}}^{(c)}$ denote high-affinity nodes (or pathways) of cell type c in BP, which composed the network of cell type-specific enriched pathways. We then divided them to determine pathway communities. Specifically, we used the python package graph-tool (DOI: 10.6084/m9.figshare.1164194.v14) v2.43 to partition these nodes according to the maximization of Newman's modularity [6]. The graph-tool adopted an agglomerative heuristic to fit the stochastic block model to achieve the community partition. We generated 100 Monte Carlo (MC) partitions and aligned them with a common community labelling. We controlled the lower limit of the number of communities as [0.05 * number of seed nodes]. Let $p\in hP_{\text{BP},t}^{(c)}$ denote pathways in community t of cell type c in BP, the community-level significance is determined by

$$\text{Significance}_{\text{BP},t}^{(c)} = \text{mean}\left(\left\{\min(4, -\log_{10}(P\text{-value}_{p,c}^{(u)}))\right\}_{p \in hP_{\text{BP},t}^{(c)}}\right) \tag{23}$$

Here, equation (23) denotes the significance of community t of cell type c in BP. We defined communities with significance greater than $-\log_{10}(\text{Active}P)$ (default 2) as active (or significant) communities. We further filtered out communities with a low number of pathways by the average number of pathways contained by significant communities. Prioritized top communities were selected based on significance rankings. In this study, we manually summarize the topic of a community to maximally cover the semantics of pathways within the community. It should be noted that all results of this part reported in this paper were based on BP because MF contained a low number of pathways. For the differentially expressed gene (DEG) list generated from the Conrow-Graham et al. *Adnp* KO dataset [7], we set Active$P$ to 0.001 because there were more than 500 original active pathways in microglia.

Step 7: ORA-based pathway analysis in CellGO

CellGO included the function of conventional pathway enrichment analysis similar to gProfileR [8-10]. Given a set of genes denoted by $g \in G^{(u)}$, and let $G^{(p)}$ denote the collection of all annotated genes of a term p and all of its child terms. We next used the python package scipy [5] v1.5.4 to perform Fisher's exact test to evaluate the pathway-level significance on a given gene list:

$$P\text{-value}_{p}^{(u)} = \text{Fisher's exact test}\left(\begin{bmatrix}\text{length}(G^{(u)} \cap G^{(p)}) & \text{length}(G^{(u)} - G^{(u)} \cap G^{(p)}) \\ \text{length}(G^{(p)}) & \text{length}(G - G^{(p)})\end{bmatrix}, \atop \text{alternative} = \text{'greater'}\right) \tag{24}$$

These results were output simultaneously with cell type-specific P-values.

3 Differences of the VNN between CellGO and Dcell

Compared with Dcell [4], the VNN in the modeling phase of CellGO has made the following changes and upgrades: (1) Dcell is designed to predict phenotype (such as the relative growth rate of yeast) from genotype (a binary vector indicating gene deletions). However, CellGO trains the VNN by inputting a single-cell expression matrix to predict identities of cells, then CellGO scores the cell type-specific activities of gene-term pairs by assessing the impact of gene expression perturbation within a pathway on cell identity prediction. (2) CellGO trains each subtree-based VNN individually and collects cell type-specific scores of all gene-term within it. (3) Compared to Dcell, CellGO uses more neurons to capture multi-functions of GO terms. (4) We optimized the network architecture of Dcell to reduce the VRAM consumption significantly. In CellGO, each GO term only inputs the expression of its annotated genes rather than the expression of all genes. (5) We implemented CellGO using the python package torch (https://github.com/pytorch/pytorch), while Dcell is implemented using the Torch7 library (https://github.com/torch/torch7).

4 Collection and processing of single-cell RNA-seq datasets

71 brain datasets

Human and mouse brain scRNA-seq or snRNA-seq datasets [11-39] were collected from publicly available resources, including GEO (https://www.ncbi.nlm.nih.gov/geo/), UCSC Cell Browser (https://cells.ucsc.edu/), Broad Single Cell Portal (https://singlecell.broadinstitute.org/), Human Cell Atlas (https://data.humancellatlas.org/), Allen Brain Atlas (http://celltypes.brain-map.org/download), DropViz (http://dropviz.org/), and Synapse (https://www.synapse.org/). We only selected datasets in which both the processed expression data and pre-assigned cell labels were available. Processed expression data could be read count and UMI count. It could also be CPM, RPKM, TPM, and log-transformation of these forms. We did not perform any pre-analysis on FASTQ files.

A total of 71 datasets were curated and processed separately to make up the proposed database according to the following steps: (i) When the obtained value was read count or UMI count, it will be normalized by the

NormalizeData function from the R package Seurat [40] v4.3.0 with default parameters to allow correction for the total number of reads per cell and log-transformation. Other values, such as CPM, RPKM, and TPM, were log2-transformed with pseudo-count one unless the expression data was already log-transformed. (ii) Quality control (QC) of cells was performed as described in the original study unless the obtained dataset was already QC-ed. (iii) Cells with uninformative cell type labels (e.g., 'unclassified') defined as outliers in the original study were excluded. We also removed unimportant and low-quantity cell types in the brain, such as T cells, endothelial cells, pericytes, and vascular leptomeningeal cells. We only retained cells of the control group. (iv) We manually aligned cell type labels annotated in the original study to the unified terminology to ensure consistency between datasets. 61 out of the 71 datasets were re-annotated at major-type-level, including excitatory neurons, inhibitory neurons, radial glias, intermediate progenitor cells, etc. Other ten datasets, which included only one major cell type, were re-annotated at subtype-level, such as excitatory neuron subtypes, including L4 IT, L5/6 IT, L6b, etc. (v) Re-annotated cell types with the number of cells less than 100 were removed. For each dataset, we exported the re-processed expression matrix and the cell identity list as two CSV files and took them as input to the modeling phase of CellGO. Details about each dataset, such as references, original download URLs, species, anatomical areas, and included cell types, are reported in Supplementary Table S1.

One PBMC dataset

The human PBMC dataset [41] was collected from UCSC Cell Browser (https://cells.ucsc.edu/), and both the log-transformed UMI count data that has been QC-ed and pre-assigned cell labels were downloaded. We only retained B cells, CD4+ T cells, CD8+ T cells, Gamma-Delta T cells, monocytes, and natural killer cells. Details about the dataset are reported in Supplementary Table S1.

5 Identification of predefined cell type-specific pathways

We collected marker genes of five cell types from the McKenzie et al. study [42]. These five cell types included neurons, oligodendrocytes, oligodendrocyte precursor cells, astrocytes, and microglia (Supplementary Table S2). The top 50 marker genes were selected for each cell type based on the reported rankings. We then took the marker genes of each cell type as input to perform conventional pathway enrichment analysis using CellGO. Predefined cell type-specific pathways were selected for each cell type according to the top 50 pathways ranked by *P*-values (Supplementary Table S3).

6 Collection of experimental DEG lists

We first considered five experimental datasets, including the bulk RNA sequencing data for both the experimental and control groups (Supplementary Table S6). We next screened the top 100 differentially expressed genes based on *P*-value rankings from the supplementary data of corresponding references. Differentially expressed genes were achieved by differential gene expression analysis between the experimental and control groups. For the Conrow-Graham et al. *Adnp* KO dataset [7], the supplementary data only reported up-regulated genes in the KO group.

7 Comparison with other tools

We compared our method with two existing methods: gProfileR [8-10] and scMappR [43]. We applied these two methods to DEG lists from Smith et al. [44], Runge et al. [45], and Wang et al. [46]. gProfileR takes a set of genes as input and returns *P*-values of pathways. We used the gprofiler function from the R package gProfileR v0.7.0 with default parameters to analyze the collected DEG lists and obtained FDR-corrected *P*-values. scMappR requires not only a DEG list but also the *P*-values and log-transformed fold changes of DEGs, the bulk expression matrix, and the single-cell gene signature matrix as input, then scMappR returns cell type-specific *P*-values of pathways. The single-cell gene signature matrix was generated based on dataset 47 using the seurat_to_generes and generes_to_heatmap functions from the R package scMappR v1.0.9 with default parameters. The adopted type of the single-cell gene signature matrix was 'OR' (odds ratios). The *P*-values and log-transformed fold changes matching the collected DEG lists were obtained from the supplementary data of corresponding references (Supplementary Table S6). The bulk expression matrices were collected from GEO (Data Availability). We next took them to the scMappR_and_pathway_analysis function with default parameters and obtained cell type-specific FDR-corrected *P*-values. It should be noted that we

only retained pathways with the domain BP or MF.

8 Benchmarking cell type-specific functional inference using putative gold standard pathways

To more powerfully demonstrate the ability of CellGO to accurately infer cell type-specific pathways compared to scMappR, we constructed the putative gold standard cell type-specific pathways based on reported experimental validations. Specifically, for the DEG lists from Smith et al. [44], we first extracted the keywords from existing validation results. These keywords are 'ATP', 'mitochondrial', 'amino', 'amine', and 'metabolic'. Next, the gold standard astrocyte-specific pathway is defined as a pathway that semantically contains any one of these keywords. Lastly, we counted the number of active gold standard pathways per cell type in CellGO, as well as the number of significant gold standard pathways per cell type in scMappR. Notably, CellGO indicated the largest number of active gold standard pathways in astrocytes (Supplementary Figure S5A), aligning with previous experimental reports. However, scMappR showed that ExN, OPC, astrocytes, and microglia had the same number of significant gold standard pathways.

In the case of the DEG lists from Runge et al. [45], the keywords for gold standard ExN-specific pathways are 'migration', 'synapse', 'membrane potential', and 'ion'. CellGO indicated the largest number of active gold standard pathways in ExN (Supplementary Figure S5B), consistent with previous findings. However, scMappR showed that astrocytes owned the largest number of significant gold standard pathways.

For the DEG lists from Wang et al. [46], the keywords for gold standard microglia-specific pathways are 'immune', 'cell cycle', and 'catabolic'. CellGO indicated the largest number of active gold standard pathways in microglia (Supplementary Figure S5C). While scMappR also revealed microglia had the largest number of significant gold standard pathways, other cell types also enriched a set number of pathways. Overall, these results strongly demonstrate that CellGO more accurately predicts cell type-specific pathways than scMappR.

9 Application of CellGO on more gene knockout datasets

We used CellGO to analyze the differentially expressed genes generated by the other two experimental datasets [7,47]. For the top 100 differentially expressed genes between *Adnp* KO and WT mice [7], CellGO found that microglia had the highest activation according to the number of active pathways (Supplementary Figure S6B). Noteworthily, top communities of microglia represented topics related to metabolic and immune system processes, stimulus responses, signal transduction, and reproductive processes (Supplementary Table S8). More specifically, organic substance metabolic process, immune response-inhibiting signal transduction, positive regulation of leukocyte differentiation, and reproductive process were found in these communities (Supplementary Figure S6B), with *Hexb* (a gene encoding the beta subunit of the lysosomal enzyme beta-hexosaminidase that breaks down fatty compounds and complex sugars), *Lyn* (a gene encoding the tyrosine protein kinase that involves in immune response-regulating signaling pathways), and *Inpp5d* (a gene encoding the protein that functions as a negative regulator of myeloid cell proliferation and survival) identified as the MAGs in these pathways. These findings are consistent with previous studies [7] showing that *Adnp* KO leads to pro-inflammatory and pro-phagocytic phenotype and a prominent increased number of microglia.

For the top 100 differentially expressed genes between neuron-specific *Wwox* knockdown and WT mice [47], CellGO discovered that oligodendrocytes showed the highest activation (Supplementary Figure S6C). Notably, top communities of oligodendrocytes represented topics related to cell development, metabolic processes, homeostatic processes, signaling, and ion transport (Supplementary Table S8). More concretely, glial cell development, cell differentiation, transmission of nerve impulse, and positive regulation of calcium ion transmembrane transport were found in these communities (Supplementary Figure S6C), with *Plp1* (a gene encoding a transmembrane proteolipid protein that is the predominant component of myelin and plays a role in oligodendrocyte development and axonal survival) and *Nfasc* (a gene encoding the protein functions in neurite outgrowth, neurite fasciculation, and organization of the axon initial segment) predicted as the MAGs in these pathways. These findings align with a previous study [47] indicating that

Adnp knockdown in neurons reduced oligodendrocyte maturation and induced hypomyelination and impaired axonal conductivity. These results again demonstrated that CellGO could be used effectively to infer functionally relevant cell types and cell type-specific pathways.

10 Application of CellGO on risk genes of systemic lupus erythematosus

In order to prove that CellGO can be used to analyze the risk genes of multi-locus genetic diseases in other systems except the nervous system, we used the human PBMC dataset [41] to run the modeling phase of CellGO, and leveraged 120 GWAS risk genes of systemic lupus erythematosus (SLE) [48] to try to elucidate the blood cell types relevant to SLE, as well as their cell type-specific pathogenic mechanisms.

Our analysis identified B cells as the PBMC cell type most associated with SLE, along with T cells showing a high degree of enrichment (Supplementary Figure S8A), consistent with the known knowledge that SLE is mainly characterized by the excessive production of pathogenic autoantibodies by the B cells [49,50], and T cells play a significant role inducing B cells to generate autoantibodies [49]. The top communities of B cells were linked to topics related to environmental stimulus responses, protein kinase signaling, tolerance induction to self antigen, and macromolecule biosynthetic processes (Supplementary Figure S8B and Supplementary Table S10). These topics correspond to the abnormal secretion of autoantibodies and responses to antigens by B cells, and the known effect of protein kinase in enhancing B cell activation and secretion of immunoglobulin M , during SLE [49]. Moreover, *BANK1*, the gene encoded the B cell-specific scaffold protein, was identified as the MAG of pathways in multiple communities (Supplementary Figure S8B). This finding aligns with its known role in promoting B cell activation and inflammation [51], representing the prominent features of SLE. In conclusion, our results once again demonstrated the ability of CellGO to understand complex multi-gene diseases.

11 Embedding a set of pathways to the semantic space

Given a set of pathways denoted by $p \in P^{(u)}$, where P represents the collection of every pathway p in the pruned GO graph. The probability of a pathway is defined as

$$\rho_p = \frac{D_p}{D_{r_p}} \tag{25}$$

Here, D_p denotes the number of p and all of its children. r_p denotes the top-level pathway of p. The top-level pathway can only be one of 'biological_process' (GO:0008150) and 'molecular_function' (GO:0003674). Let $\rho_{\min}(p_1, p_2)$ denote the minimum probability of the common ancestor pathways of p_1 and p_2, we next used the Lin method [52] to determine the similarity between the two pathways:

$$Similarity(p_1, p_2) = \frac{2 * \rho_{\min}(p_1, p_2)}{\rho_{p_1} + \rho_{p_2}} \tag{26}$$

We computed the similarity for all pathway-pathway pairs in $P^{(u)}$, which composed the similarity matrix $\mathbb{R}^{U \times U}$, where U is the number of pathways in $P^{(u)}$. We next used the umap function from the R package umap [53] v0.2.9.0 with default parameters to reduce the length of the second dimension of the similarity matrix to two. Since BP and MF are two non-interconnected domains, we only mapped the BP pathways to the two-dimensional semantic space.

12 Hyperparameter selection of CellGO

In the modeling phase of CellGO, there are four hyperparameters that involve in the neural network training. These hyperparameters include the learning rate, the l_2 norm regularization factor, the training epoch number, and the balance coefficient α in equation (5). We referred to the hyperparameters adopted by Dcell [4] and directly set the l_2 norm regularization factor to 0.001 and the balance coefficient α to 0.3.

For the other two hyperparameters, the learning rate and the training epoch number, we performed hyperparameter grid search based on three evaluation metrics: the classification accuracy, the reproducibility, and the running time. The larger the first two and the smaller the last one, the better. Specifically, the classification accuracy is defined as the median cell type classification accuracy of 4,168 subtree-based VNNs; the reproducibility is defined as the average value of pairwise Spearman's rank correlations of GTSs between replicates of the same cell type, and these correlations were calculated on gene-term pairs with GTSs ranked in the top 5% in either group, the replicate number is two; the running time is defined as the CPU time spent in the modeling phase. We trained CellGO on dataset 17 using nine different hyperparameter combinations (learning rate = 0.0001, 0.001, and 0.005; epoch number = 10, 15, and 20). We found that the classification accuracy increases with the learning rate, however, the reproducibility decreases with the learning rate (Supplementary Figure S10). To balance these two evaluation metrics, we chose the learning rate at 0.001. In addition, we discovered that the classification accuracy and the reproducibility slightly increase with the epoch number, whereas the running time moderately increases with the epoch number (Supplementary Figure S10). To balance these three evaluation metrics, we set the epoch number to 15. Together, we finally determined four hyperparameters for the modeling phase: l_2 norm regularization factor=0.001, balance coefficient $\alpha = 0.3$, learning rate = 0.001, and epoch number = 15.

In the analysis phase of CellGO, there is no hyperparameter when acquiring cell type-specific active pathways given a single gene or a gene set, when identifying the cell type most activated by the input gene set, and when selecting the most active genes within pathways. Nevertheless, there is one key hyperparameter used in pathway network analysis when constructing the network of cell type-specific active pathways. Active*P* is used to determine which pathways are active, and pathways with *P*-values no more than it will be considered as active pathways and will be sent to pathway network analysis. Ideally, a smaller Active*P* will increase the reliability of the active pathways, but will reduce the number of active pathways. Here, we give our empirical strategy to choose a suitable Active*P*: Active*P* equals to 0.01 when the number of active pathways is no more than 500, otherwise Active*P* equals 0.001. We used this strategy throughout this study and found it performed well. Finally, there are some unimportant hyperparameters during the pathway network analysis, such as the number of travels starting from a seed node and the number of steps per travel in the RWR algorithm. Despite the larger these parameters will lead to more confident results, they are sufficient to complete the required analysis.

13 CellGO's imperfect performance using standardized scRNA-seq data

To investigate the analysis results of models trained on standardized scRNA-seq data, we inputted the standardized single-cell expression matrix of dataset 47 to the modeling phase, and other parameters were unchanged. This standardization procedure is performed by the ScaleData function from the R package Seurat [40] v4.3.0, using the normalized single-cell expression matrix (Supplementary Text S4).

Next, we applied CellGO to examine *Atp1a2* and *Neurod2* using single-gene analysis based on the model trained on standardized dataset 47. We found that astrocytes was the most affected cell type by *Atp1a2* perturbation (Supplementary Figure S11A). In addition, pathways with the highest GTSs in astrocytes were related to ion homeostasis and stimulus responses (Supplementary Figure S11B and Supplementary Table S12). These above findings are consistent with the results based on normalized dataset 47 (Figure 3A-B). However, our single-gene analysis revealed *Neurod2* primarily affected InN, inconsistent with the results using normalized data (Figure 3A) and the reported more functions of *Neurod2* on ExN than InN [45].

When extending the analysis to the top 100 differentially expressed genes between astrocyte-specific *Atp1a2* KO and WT mice, CellGO revealed the largest number of active pathways in astrocytes. However, some key experimentally verified pathways found by the normalized dataset 47, such as amine biosynthetic process and primary amino compound metabolic process (Figure 3C), were not active in astrocytes (Supplementary Figure S11C and Supplementary Table S12). For the analysis of the top 100 differentially expressed genes between *Neurod2* KO and WT mice, CellGO identified ExN as the cell type with the highest activation. Nevertheless, one experimentally verified pathway found by the normalized dataset 47 (Figure 3D), behavior, was not active

in excitatory or inhibitory neurons (Supplementary Figure S11D and Supplementary Table S12). Overall, these results suggested that analysis outcomes generated from the standardized scRNA-seq count data are worse than those from the normalized scRNA-seq count data.

Supplementary Reference

1. Ashburner M, Ball CA, Blake JA, *et al*. Gene Ontology: tool for the unification of biology. Nat Genet 2000; **25**:25–29.
2. The Gene Ontology Consortium, Carbon S, Douglass E, *et al*. The Gene Ontology resource: enriching a GOld mine. *Nucleic Acids Res* 2021; **49**:D325–D334.
3. Klopfenstein DV, Zhang L, Pedersen BS, *et al*. GOATOOLS: A Python library for Gene Ontology analyses. *Sci Rep* 2018; **8**:10872.
4. Ma J, Yu MK, Fong S, *et al*. Using deep learning to model the hierarchical structure and function of a cell. *Nat Methods* 2018; **15**:290–298.
5. Virtanen P, Gommers R, Oliphant TE, *et al*. SciPy 1.0: fundamental algorithms for scientific computing in Python. *Nat Methods* 2020; **17**:261–272.
6. Newman MEJ. Modularity and community structure in networks. *Proc Natl Acad Sci U S A* 2006; **103**:8577–8582.
7. Conrow-Graham M, Williams JB, Martin J, *et al*. A convergent mechanism of high risk factors ADNP and POGZ in neurodevelopmental disorders. *Brain* 2022; **145**:3250–3263.
8. Reimand J, Kull M, Peterson H, *et al*. g:Profiler—a web-based toolset for functional profiling of gene lists from large-scale experiments. *Nucleic Acids Res* 2007; **35**:W193–W200.
9. Reimand J, Arak T, Adler P, *et al*. g:Profiler—a web server for functional interpretation of gene lists (2016 update). *Nucleic Acids Res* 2016; **44**:W83–W89.
10. Raudvere U, Kolberg L, Kuzmin I, *et al*. g:Profiler: a web server for functional enrichment analysis and conversions of gene lists (2019 update). *Nucleic Acids Res* 2019; **47**:W191–W198.
11. Velmeshev D, Schirmer L, Jung D, *et al*. Single-cell genomics identifies cell type–specific molecular changes in autism. *Science* 2019; **364**:685–689.
12. Kamath T, Abdulraouf A, Burris SJ, *et al*. Single-cell genomic profiling of human dopamine neurons identifies a population that selectively degenerates in Parkinson's disease. *Nat Neurosci* 2022; **25**:588–595.
13. Leng K, Li E, Eser R, *et al*. Molecular characterization of selectively vulnerable neurons in Alzheimer's disease. *Nat Neurosci* 2021; **24**:276–287.
14. Franjic D, Skarica M, Ma S, *et al*. Transcriptomic taxonomy and neurogenic trajectories of adult human, macaque, and pig hippocampal and entorhinal cells. *Neuron* 2022; **110**:452-469.e14.
15. Hodge RD, Bakken TE, Miller JA, *et al*. Conserved cell types with divergent features in human versus mouse cortex. *Nature* 2019; **573**:61–68.
16. Nagy C, Maitra M, Tanti A, *et al*. Single-nucleus transcriptomics of the prefrontal cortex in major depressive disorder implicates oligodendrocyte precursor cells and excitatory neurons. *Nat Neurosci* 2020; **23**:771–781.
17. Ayhan F, Kulkarni A, Berto S, *et al*. Resolving cellular and molecular diversity along the hippocampal anterior-to-posterior axis in humans. *Neuron* 2021; **109**:2091-2105.e6.
18. Jäkel S, Agirre E, Mendanha Falcão A, *et al*. Altered human oligodendrocyte heterogeneity in multiple sclerosis. *Nature* 2019; **566**:543–547.
19. Schirmer L, Velmeshev D, Holmqvist S, *et al*. Neuronal vulnerability and multilineage diversity in multiple sclerosis. *Nature* 2019; **573**:75–82.
20. Bakken TE, Jorstad NL, Hu Q, *et al*. Comparative cellular analysis of motor cortex in human, marmoset and mouse. *Nature* 2021; **598**:111–119.
21. Kihara Y, Zhu Y, Jonnalagadda D, *et al*. Single-Nucleus RNA-seq of Normal-Appearing Brain Regions in Relapsing-Remitting vs. Secondary Progressive Multiple Sclerosis: Implications for the Efficacy of Fingolimod. *Front Cell Neurosci* 2022; **16**:918041.
22. Fullard JF, Lee H-C, Voloudakis G, *et al*. Single-nucleus transcriptome analysis of human brain

immune response in patients with severe COVID-19. *Genome Med* 2021; **13**:118.
23. Morabito S, Miyoshi E, Michael N, *et al*. Single-nucleus chromatin accessibility and transcriptomic characterization of Alzheimer's disease. *Nat Genet* 2021; **53**:1143–1155.
24. Ma S, Skarica M, Li Q, *et al*. Molecular and cellular evolution of the primate dorsolateral prefrontal cortex. *Science* 2022; **377**:eabo7257.
25. Bhaduri A, Andrews MG, Mancia Leon W, *et al*. Cell stress in cortical organoids impairs molecular subtype specification. *Nature* 2020; **578**:142–148.
26. Bhaduri A, Sandoval-Espinosa C, Otero-Garcia M, *et al*. An atlas of cortical arealization identifies dynamic molecular signatures. *Nature* 2021; **598**:200–204.
27. Yu Y, Zeng Z, Xie D, *et al*. Interneuron origin and molecular diversity in the human fetal brain. *Nat Neurosci* 2021; **24**:1745–1756.
28. Trevino AE, Müller F, Andersen J, *et al*. Chromatin and gene-regulatory dynamics of the developing human cerebral cortex at single-cell resolution. *Cell* 2021; **184**:5053-5069.e23.
29. Fan X, Fu Y, Zhou X, *et al*. Single-cell transcriptome analysis reveals cell lineage specification in temporal-spatial patterns in human cortical development. *Sci Adv* 2020; **6**:eaaz2978.
30. Yuan W, Ma S, Brown JR, *et al*. Temporally divergent regulatory mechanisms govern neuronal diversification and maturation in the mouse and marmoset neocortex. *Nat Neurosci* 2022; **25**:1049–1058.
31. Ximerakis M, Lipnick SL, Innes BT, *et al*. Single-cell transcriptomic profiling of the aging mouse brain. *Nat Neurosci* 2019; **22**:1696–1708.
32. Cheng S, Butrus S, Tan L, *et al*. Vision-dependent specification of cell types and function in the developing cortex. *Cell* 2022; **185**:311-327.e24.
33. Hajdarovic KH, Yu D, Hassell L-A, *et al*. Single-cell analysis of the aging female mouse hypothalamus. *Nat Aging* 2022; **2**:662–678.
34. Saunders A, Macosko EZ, Wysoker A, *et al*. Molecular Diversity and Specializations among the Cells of the Adult Mouse Brain. *Cell* 2018; **174**:1015-1030.e16.
35. Yao Z, van Velthoven CTJ, Nguyen TN, *et al*. A taxonomy of transcriptomic cell types across the isocortex and hippocampal formation. *Cell* 2021; **184**:3222-3241.e26.
36. Chen R, Blosser TR, Djekidel MN, *et al*. Decoding molecular and cellular heterogeneity of mouse nucleus accumbens. *Nat Neurosci* 2021; **24**:1757–1771.
37. Di Bella DJ, Habibi E, Stickels RR, *et al*. Molecular logic of cellular diversification in the mouse cerebral cortex. *Nature* 2021; **595**:554–559.
38. Loo L, Simon JM, Xing L, *et al*. Single-cell transcriptomic analysis of mouse neocortical development. *Nat Commun* 2019; **10**:134.
39. La Manno G, Siletti K, Furlan A, *et al*. Molecular architecture of the developing mouse brain. *Nature* 2021; **596**:92–96.
40. Hao Y, Hao S, Andersen-Nissen E, *et al*. Integrated analysis of multimodal single-cell data. Cell 2021; **184**:3573-3587.e29.
41. Guo C, Li B, Ma H, et al. Single-cell analysis of two severe COVID-19 patients reveals a monocyte-associated and tocilizumab-responding cytokine storm. *Nat Commun* 2020; **11**:3924.
42. McKenzie AT, Wang M, Hauberg ME, *et al*. Brain Cell Type Specific Gene Expression and Co-expression Network Architectures. *Sci Rep* 2018; **8**:8868.
43. Sokolowski DJ, Faykoo-Martinez M, Erdman L, *et al*. Single-cell mapper (scMappR): using scRNA-seq to infer the cell-type specificities of differentially expressed genes. *NAR Genom Bioinf* 2021; **3**:lqab011.
44. Smith SE, Chen X, Brier LM, *et al*. Astrocyte deletion of α2-Na/K ATPase triggers episodic motor paralysis in mice via a metabolic pathway. *Nat Commun* 2020; **11**:6164.
45. Runge K, Mathieu R, Bugeon S, *et al*. Disruption of NEUROD2 causes a neurodevelopmental syndrome with autistic features via cell-autonomous defects in forebrain glutamatergic neurons. *Mol Psychiatry* 2021; **26**:6125–6148.
46. Wang C, Fan L, Khawaja RR, *et al*. Microglial NF-κB drives tau spreading and toxicity in a mouse model of tauopathy. *Nat Commun* 2022; **13**:1969.

47. Repudi S, Steinberg DJ, Elazar N, *et al*. Neuronal deletion of Wwox, associated with WOREE syndrome, causes epilepsy and myelin defects. *Brain* 2021; **144**:3061–3077.
48. Wang Y-F, Zhang Y, Lin Z, et al. Identification of 38 novel loci for systemic lupus erythematosus and genetic heterogeneity between ancestral groups. *Nat Commun* 2021; **12**:772.
49. Ameer MA, Chaudhry H, Mushtaq J, et al. An Overview of Systemic Lupus Erythematosus (SLE) Pathogenesis, Classification, and Management. *Cureus* 2022; **14**:e30330.
50. Sandling JK, Pucholt P, Hultin Rosenberg L, et al. Molecular pathways in patients with systemic lupus erythematosus revealed by gene-centred DNA sequencing. *Ann Rheum Dis* 2021; **80**:109–117.
51. Bolin K, Imgenberg-Kreuz J, Leonard D, et al. Variants in BANK1 are associated with lupus nephritis of European ancestry. *Genes Immun* 2021; **22**:194–202.
52. Lin D. An Information-Theoretic Definition of Similarity. In: *Proceedings of the Fifteenth International Conference on Machine Learning.* Morgan Kaufmann Publishers Inc., 1998, 296–304.
53. McInnes L, Healy J, Melville J. UMAP: Uniform Manifold Approximation and Projection for Dimension Reduction. arXiv:1802.03426, 2018.

Supplementary Figure

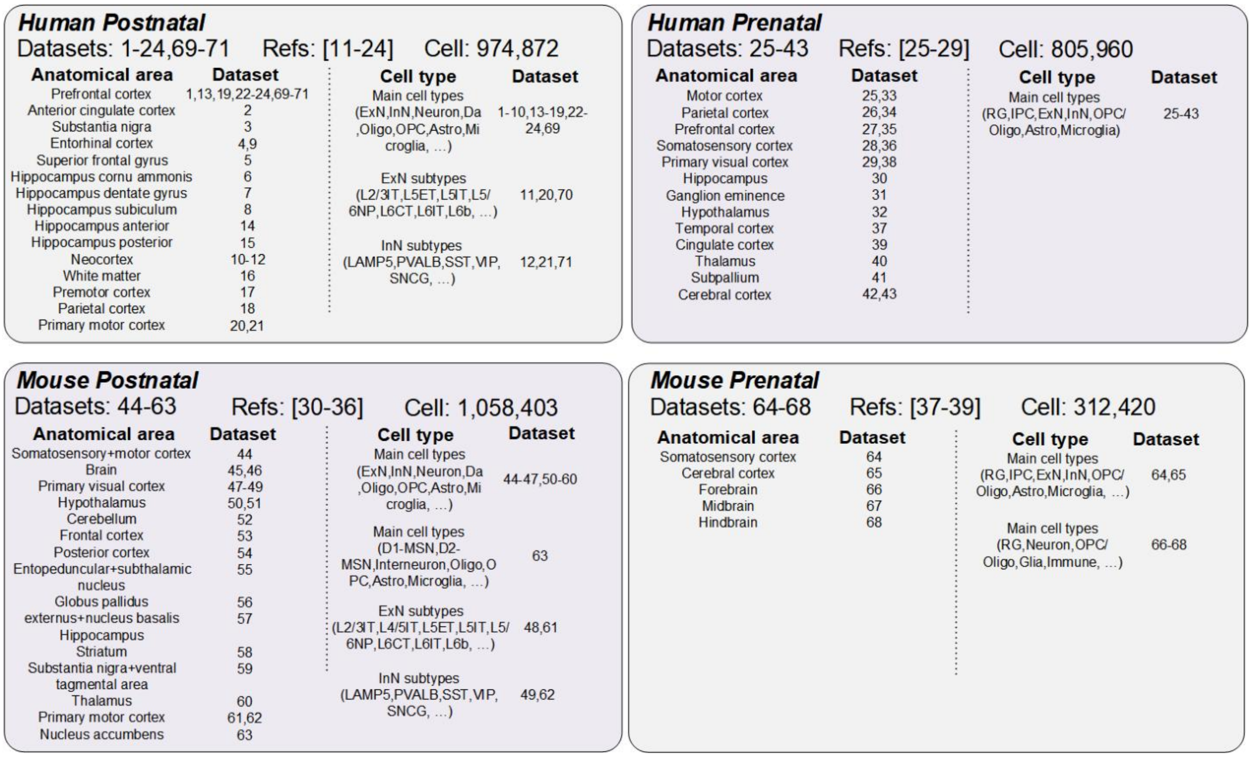

Supplementary Figure 1. Details about 71 brain cell datasets inbuilt in CellGO.
The anatomical area and included cell types per single-cell dataset are shown. More detailed description of each dataset as well as full names of cell types are in Supplementary Table S1.

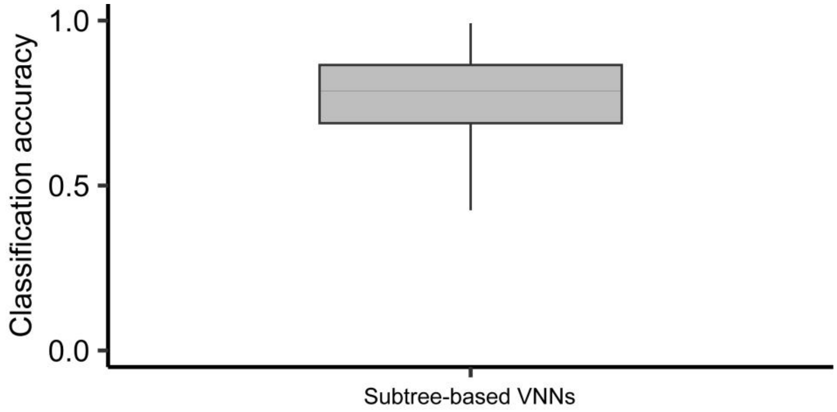

Supplementary Figure 2. The accuracy of CellGO's performance in cell type classification.
Boxplot indicating the classification accuracy of 4,168 subtree-based VNNs using dataset 1. The boxplot displays the median as the horizontal middle line, the 0.25 quantile (Q_1) and 0.75 quantile (Q_3) as the bounds of the box, and the upper and lower whiskers based on the minimum of the maximum value and $Q_3 + 1.5*IQR$, and the maximum of the minimum value and $Q_1 - 1.5*IQR$, respectively.

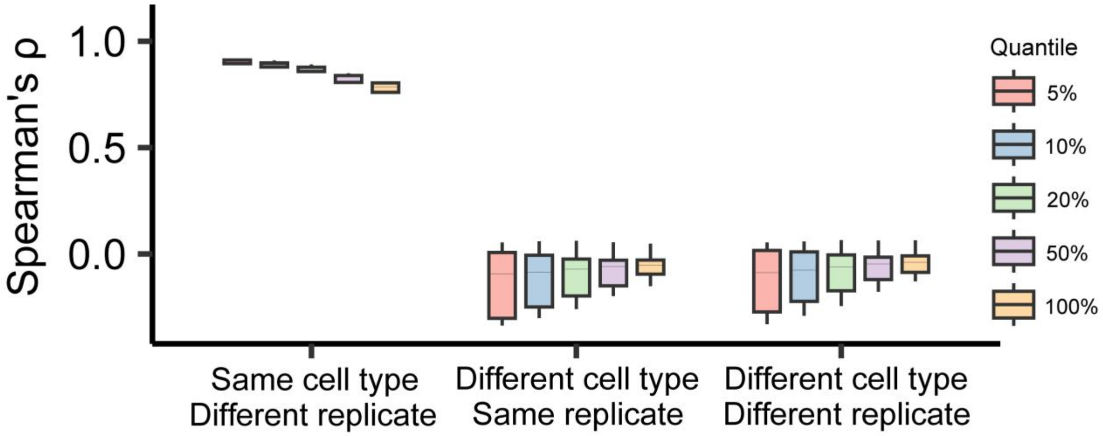

Supplementary Figure 3. Assessment of model reproducibility.
Boxplot showing pairwise Spearman's rank correlation coefficients across GTSs of the same cell type from different replicates (left), GTSs of different cell types from the same replicate (middle), and GTSs of different cell types from different replicates (right), calculated using four independent replicates of dataset 1. Spearman's correlations calculated using different quantiles of gene-term pairs were displayed. For example, 5% indicates that the correlations were calculated on gene-term pairs with GTSs ranked in the top 5% in either group. The boxplot displays the median as the horizontal middle line, the 0.25 quantile (Q_1) and 0.75 quantile (Q_3) as the bounds of the box, and the upper and lower whiskers based on the minimum of the maximum value and $Q_3 + 1.5*IQR$, and the maximum of the minimum value and $Q_1 - 1.5*IQR$, respectively.

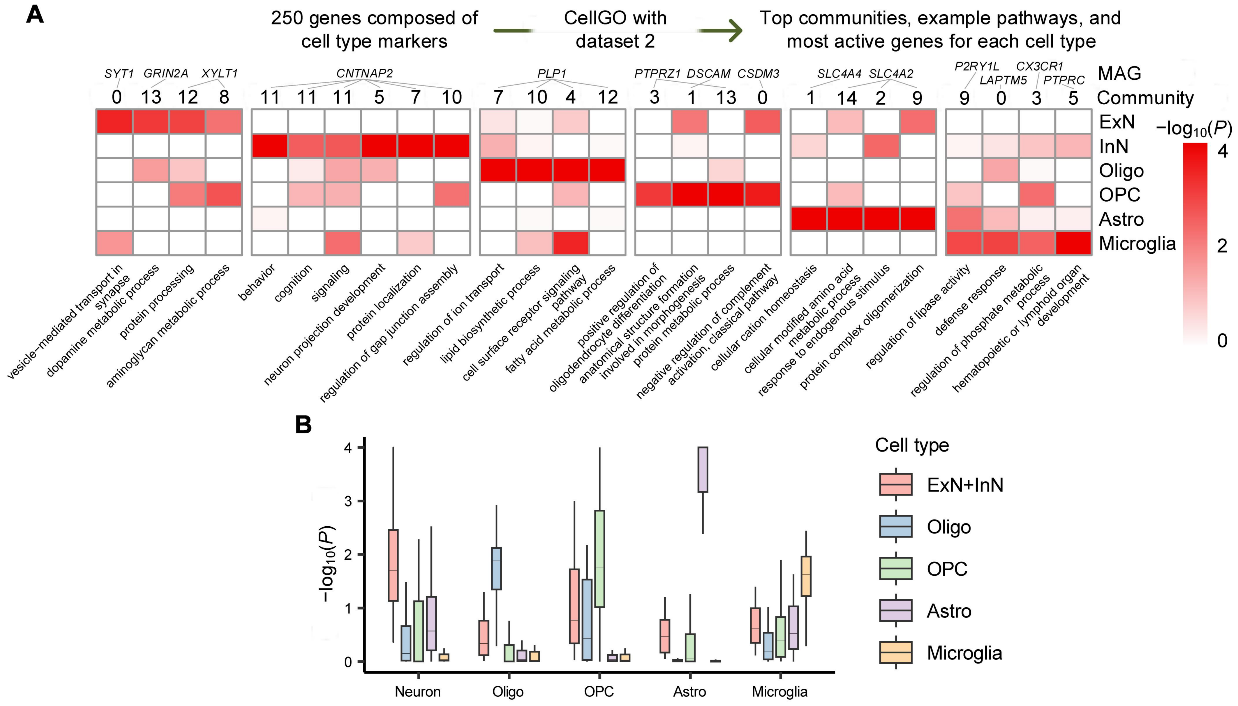

Supplementary Figure 4. Application of CellGO to capture cell type-specific signatures using dataset 2. (**A**) Top four communities, example pathways in these communities, and MAGs of these pathways for each cell type identified by CellGO testing 250 cell type markers using dataset 2. Heatmap colors denote the cell type-specific log-transformed *P*-values of example pathways in the top four communities per cell type. The community index and the MAG of each pathway are shown above the heatmap.

(**B**) Box plot showing cell type-specific log-transformed *P*-values from CellGO for predefined cell type-specific pathways. The boxplot displays the median as the horizontal middle line, the 0.25 quantile (Q_1) and 0.75 quantile (Q_3) as the bounds of the box, and the upper and lower whiskers based on the minimum of the maximum value and $Q_3 + 1.5*IQR$, and the maximum of the minimum value and $Q_1 - 1.5*IQR$, respectively.

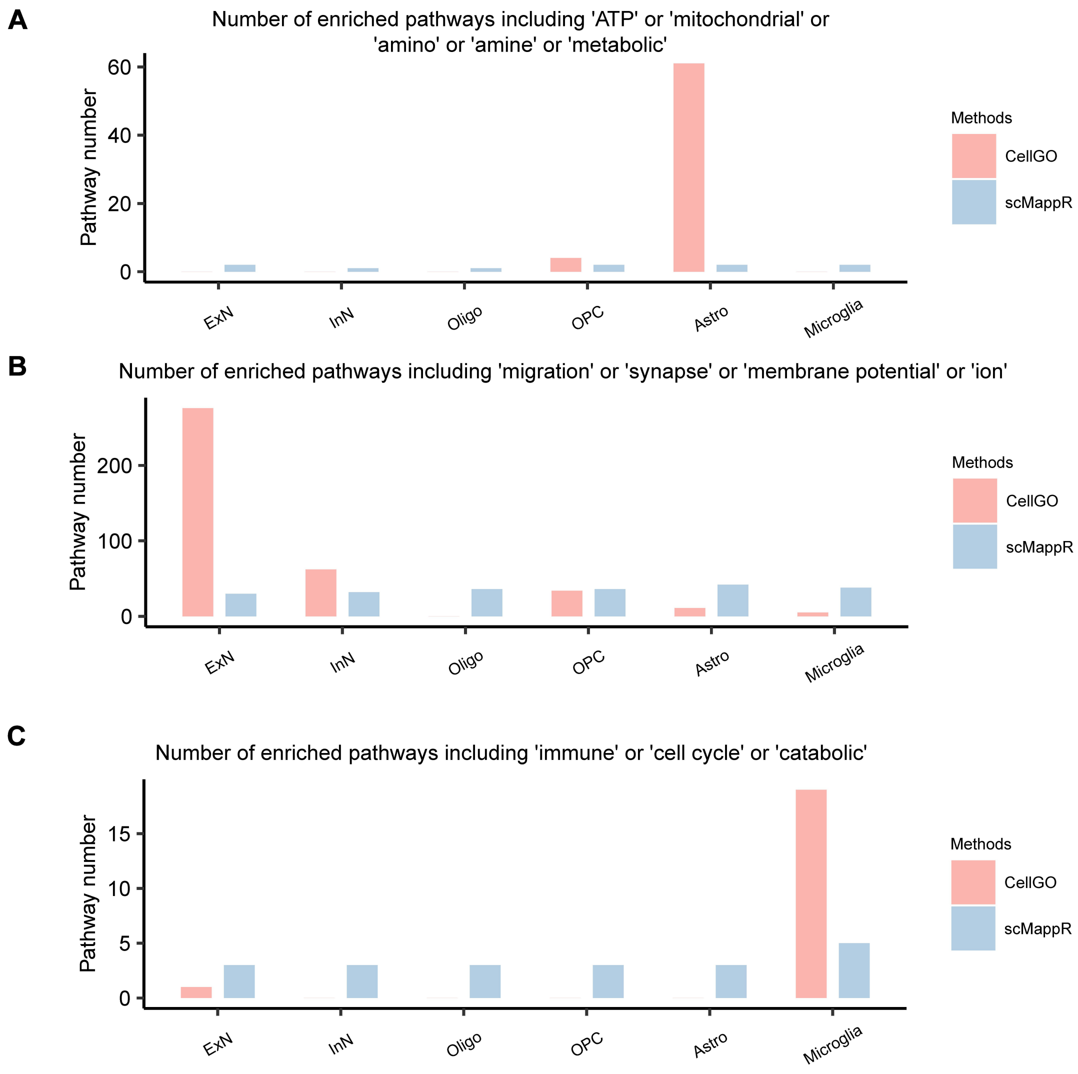

Supplementary Figure 5. Enrichment of putative gold standard pathways.
(**A-C**) Bar plot showing the number of active gold standard pathways (*P*-value < 0.01) per cell type in CellGO, as well as the number of significant gold standard pathways (FDR-adjusted *P* < 0.05) per cell type in scMappR, when analyzing differentially expressed gene lists from (**A**) Smith et al., (**B**) Runge et al., and (**C**) Wang et al.. (**A**) The putative gold standard pathway is defined as a pathway that semantically contains 'ATP' or 'mitochondrial' or 'amino' or 'amine' or 'metabolic'. (**B**) The putative gold standard pathway is defined as a pathway that semantically contains 'migration' or 'synapse' or 'membrane potential' or 'ion'. (**C**) The putative gold standard pathway is defined as a pathway that semantically contains 'immune' or 'cell cycle' or 'catabolic'.

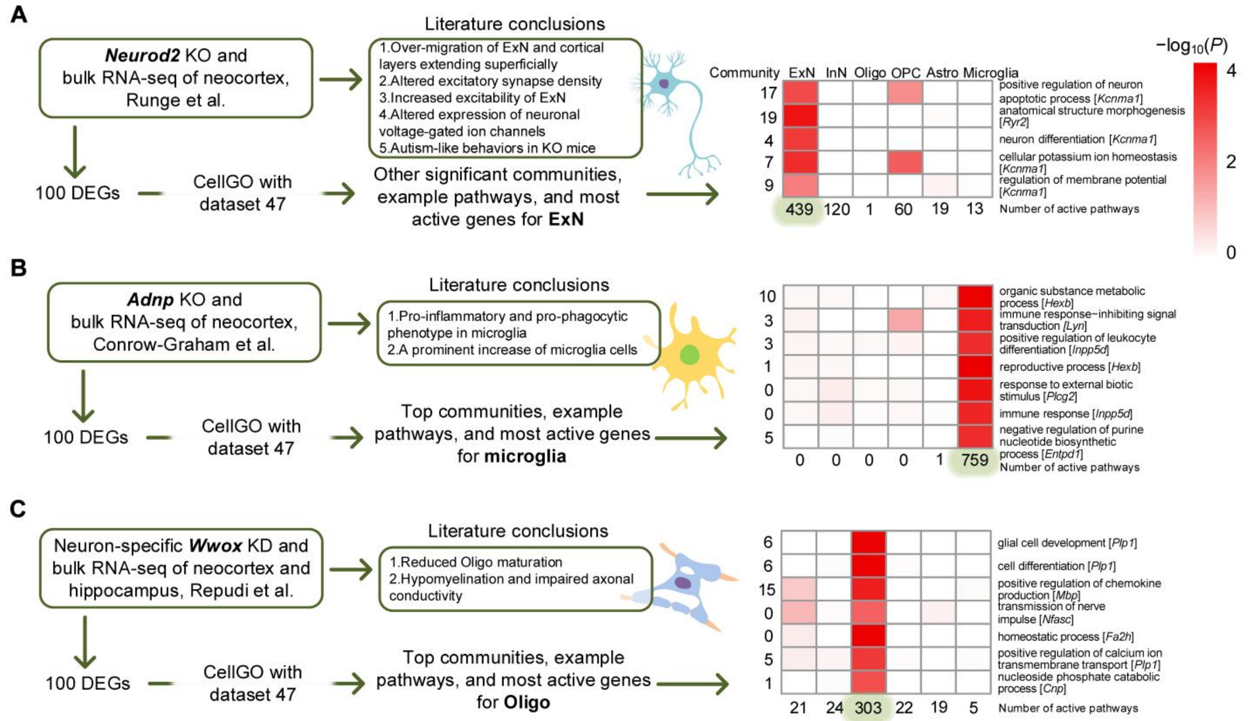

Supplementary Figure 6. Benchmarking CellGO's performance on more experimental datasets. (**A-C**), Results obtained from CellGO when analyzing differentially expressed gene lists from (**A**) Runge et al., (**B**) Conrow-Graham et al., and (**C**) Repudi et al.. The main conclusions reported in the original studies are listed as ground truth. Heatmap colors represent log-transformed *P*-values. (**A**) More significantly active pathways not in the top five communities associated with *Neurod2* are presented. (**B,C**) Example pathways in the top five communities of the most active cell type are plotted. The number of active pathways (*P*-value < 0.01) per cell type is displayed underneath the heatmap, and the MAG of each pathway is provided next to the pathway name. The community index of each pathway is shown on the left side of the heatmap.

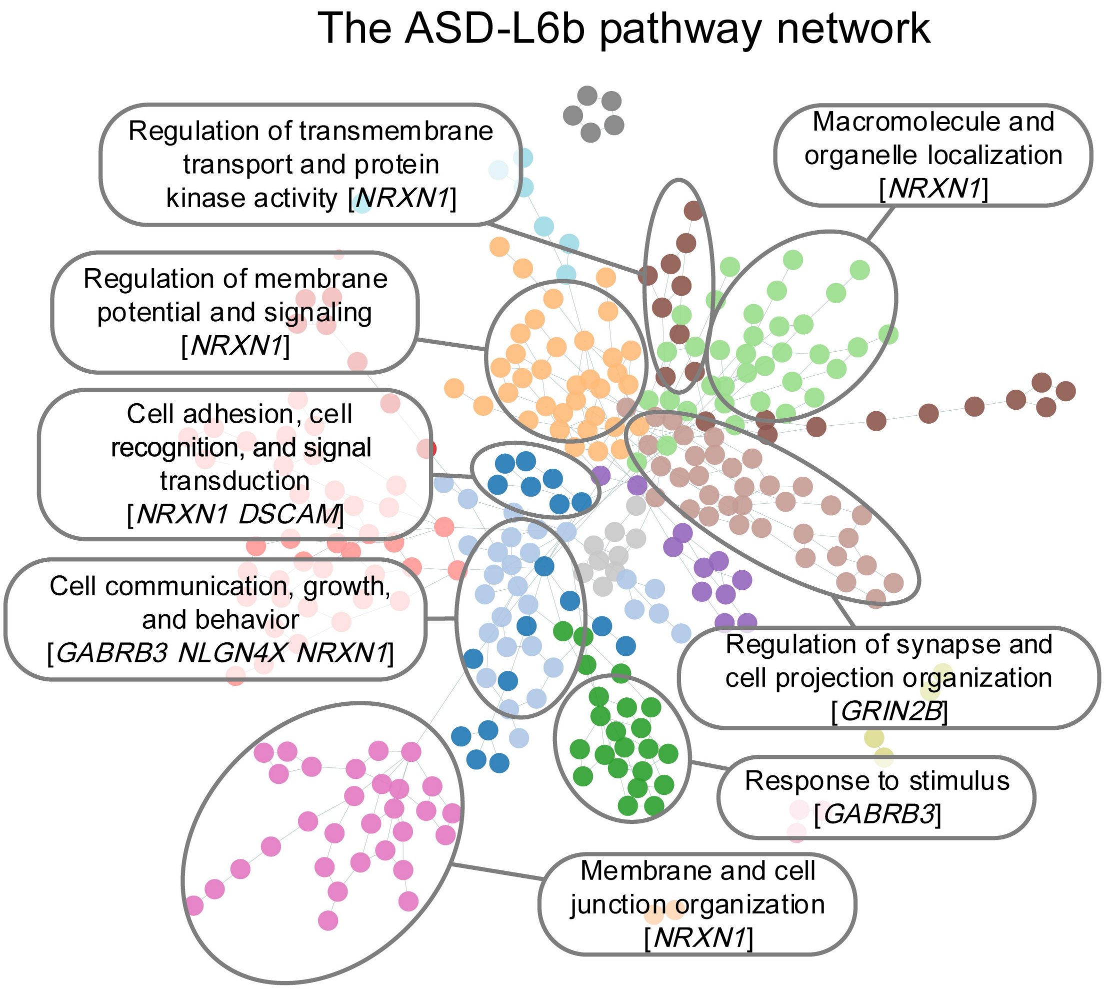

Supplementary Figure 7. The ASD-associated L6b-specific pathway network.
The pathways are colored by communities, with the top eight communities elliptically labeled and annotated with topics. MAGs of pathways with close semantics to the topic of communities are labeled.

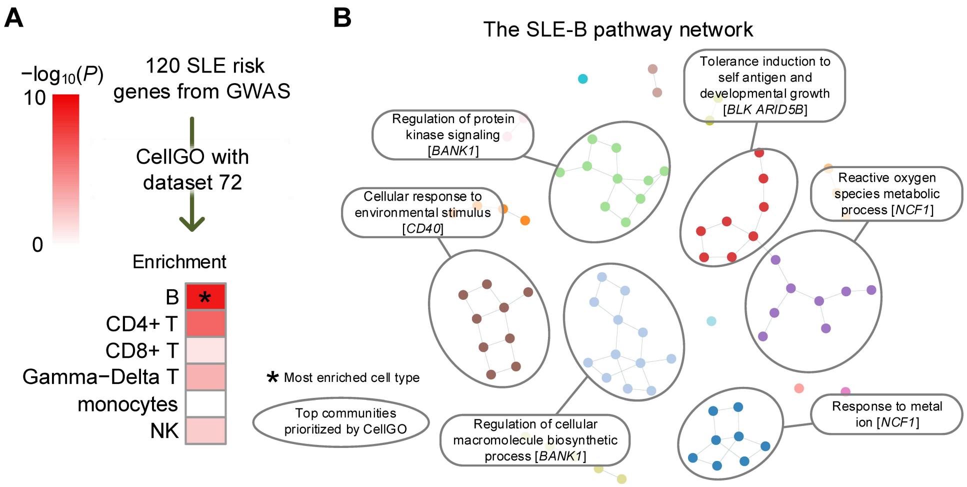

Supplementary Figure 8. Application of CellGO to risk genes of systemic lupus erythematosus.
(**A-B**) Results of CellGO analysis performed on 120 SLE risk genes using dataset 72. (**A**) The cell type enrichment of SLE-active pathways. Heatmap colors denote the log-transformed *P*-values of cell types based on K-S test of GTSs (see Methods). The asterisk denotes the most enriched cell type. (**B**) The network of SLE-active pathways in B cells. The pathways are colored by communities, with the top six communities elliptically labeled and annotated with topics. MAGs of pathways with close semantics to the topic of communities are labeled.

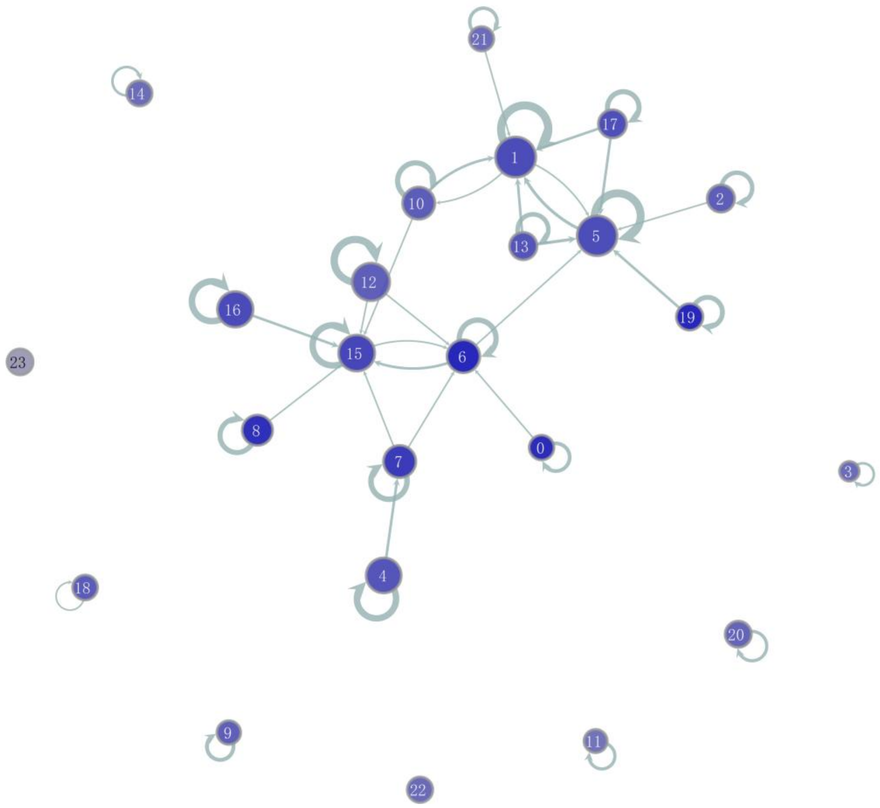

Supplementary Figure 9. The ASD-associated ExN-specific community network.
This community network is matched with Figure 4E. Each node represents a pathway community, and the community number is shown in the node; the color of each node denotes the significance of the community; the size of each node denotes the number of pathways in the community; the thickness of each edge between nodes denotes the number of connections between communities.

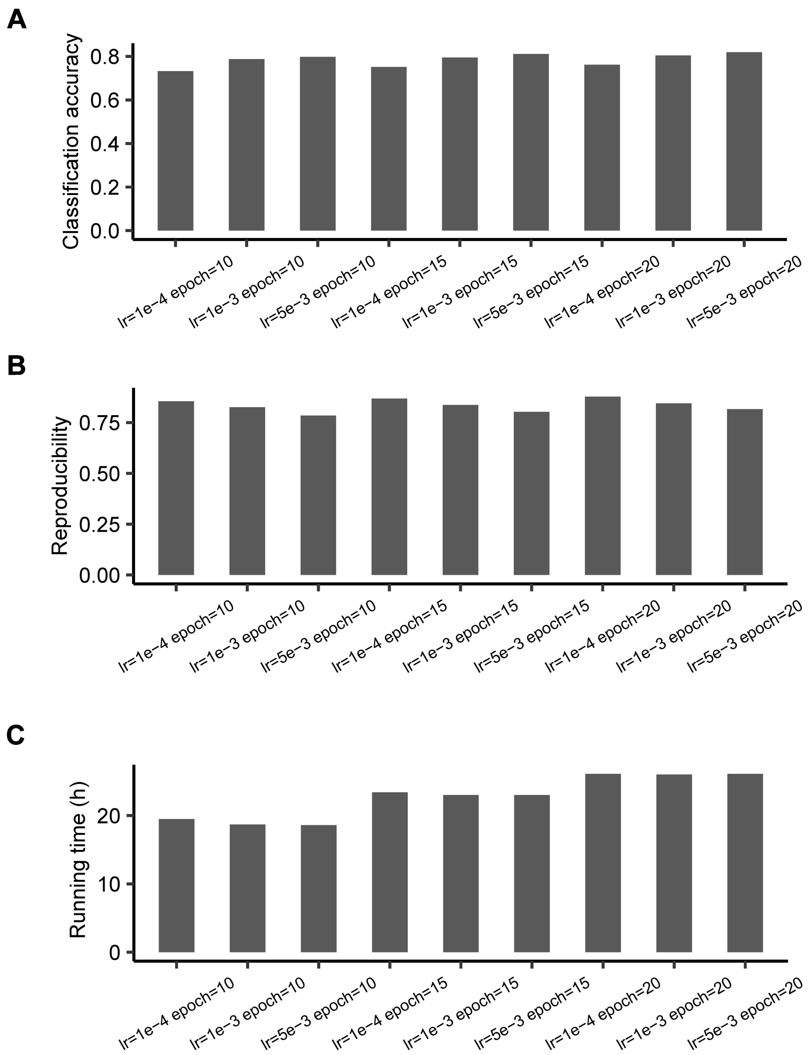

Supplementary Figure 10. Hyperparameter selection for the VNN training.
(**A-C**) We trained CellGO on dataset 17 using different hyperparameter combinations (learning rate = 0.0001, 0.001, and 0.005; epoch number = 10, 15, and 20). (**A**) Bar plot showing the median cell type classification accuracy of 4,168 subtree-based VNNs. Different hyperparameter combinations are shown under the x-axis. (**B**) Bar plot showing the average value of pairwise Spearman's rank correlation coefficients across GTSs of the same cell type from different replicates, calculated using two independent replicates of dataset 17. These correlations were calculated on gene-term pairs with GTSs ranked in the top 5% in either group. (**C**) Bar plot showing the running time of the modeling phase. lr, learning rate. h, hour.

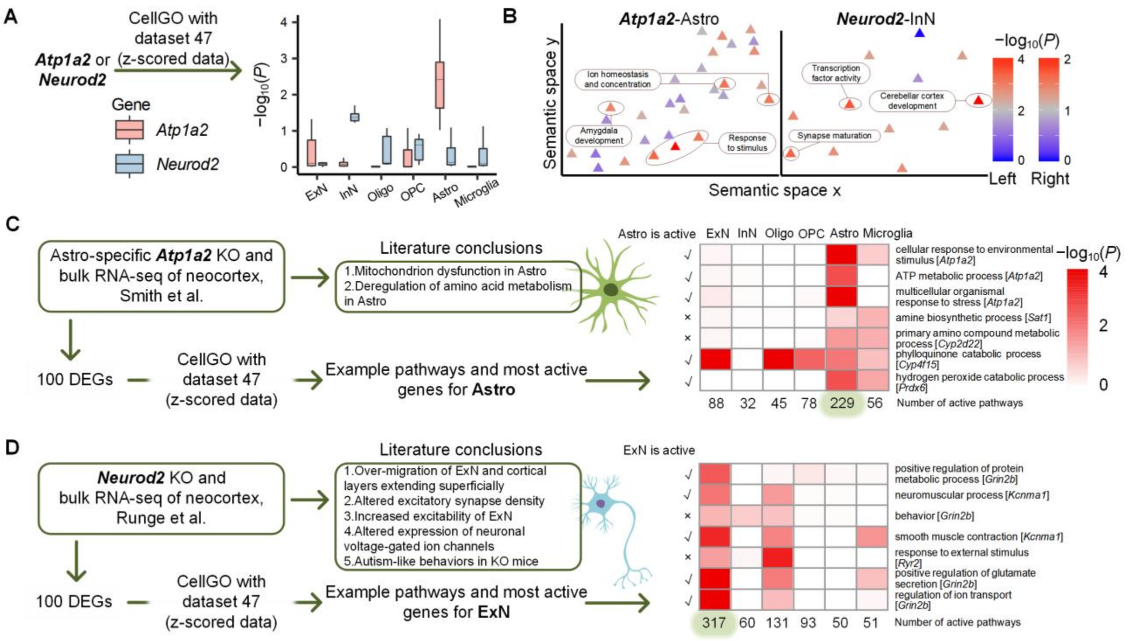

Supplementary Figure 11. CellGO's imperfect performance based on standardized scRNA-seq data.
(**A**) Boxplot illustrating the distribution of cell type-specific log-transformed *P*-values, mimicking *Atp1a2* and *Neurod2* knockout, using single-gene mode based on standardized (z-scored) dataset 47. The boxplot displays the median as the horizontal middle line, the 0.25 quantile (Q_1) and 0.75 quantile (Q_3) as the bounds of the box, and the upper and lower whiskers based on the minimum of the maximum value and $Q_3 + 1.5*IQR$, and the maximum of the minimum value and $Q_1 - 1.5*IQR$, respectively.
(**B**) Semantic map of pathways predicted to be perturbed in *Atp1a2* (left) and *Neurod2* (right) knockouts, respectively, using single-gene mode. The colors of triangles denote the log-transformed *P*-values of pathways directly connected to *Atp1a2* in astrocytes (left) or log-transformed *P*-values of pathways directly connected to *Neurod2* in InN (right). The most significant pathways are elliptically labeled and annotated with their names.
(**C**,**D**) Results obtained from CellGO when analyzing differentially expressed gene lists from (**C**) Smith et al. and (**D**) Runge et al.. The main conclusions reported in the original studies are listed as ground truth. Heatmap colors represent log-transformed *P*-values. Example pathways in Figure 3C-D are plotted. The number of active pathways (*P*-value < 0.01) per cell type is displayed underneath the heatmap, and the MAG of each pathway is provided next to the pathway name. Whether the pathway is active in the most active cell type is denoted on the left side of the heatmap.

Description of Supplementary Tables

Supplementary Table 1
Detailed information of the curated brain cell database
Supplementary Table 2
Cell type markers
Supplementary Table 3
Predefined cell type-specific pathways
Supplementary Table 4
Outputs from CellGO analyzing cell-type marker genes based on dataset 1
Supplementary Table 5
Outputs from CellGO analyzing cell-type marker genes based on dataset 2
Supplementary Table 6
Five experimental datasets and corresponding DEG lists for testing
Supplementary Table 7
CellGO outputs from single-gene-mode analysis of *Atp1a2* and *Neurod2*
Supplementary Table 8
CellGO, gProfileR, and scMappR outputs of the five DEG lists
Supplementary Table 9
Disease risk genes
Supplementary Table 10
CellGO outputs of disease risk genes
Supplementary Table 11
CellGO outputs of ASD risk genes based on four PFC datasets
Supplementary Table 12
Outputs from CellGO analyzing *Atp1a2* and *Neurod2* based on standardized dataset 47
